# Computational Insights into β-Carboline Inhibition of Monoamine Oxidase A

**DOI:** 10.3390/molecules27196711

**Published:** 2022-10-09

**Authors:** Alja Prah, Tanja Gavranić, Andrej Perdih, Marija Sollner Dolenc, Janez Mavri

**Affiliations:** 1 National Institute of Chemistry, SI-1000 Ljubljana, Slovenia; 2 Jožef Stefan Institute, SI-1000 Ljubljana, Slovenia; 3 Faculty of Pharmacy, University of Ljubljana, SI-1000 Ljubljana, Slovenia

**Keywords:** monoamine oxidase, β-carbolines, depression, linear interaction energy

## Abstract

Monoamine oxidases (MAOs) are an important group of enzymes involved in the degradation of neurotransmitters and their imbalanced mode of action may lead to the development of various neuropsychiatric or neurodegenerative disorders. In this work, we report the results of an in-depth computational study in which we performed a static and a dynamic analysis of a series of substituted β-carboline natural products, found mainly in roasted coffee and tobacco smoke, that bind to the active site of the MAO-A isoform. By applying molecular docking in conjunction with structure-based pharmacophores and molecular dynamics simulations coupled with dynamic pharmacophores, we extensively investigated the geometric aspects of MAO-A binding. To gain insight into the energetics of binding, we used the linear interaction energy (LIE) method and determined the key anchors that allow productive β-carboline binding to MAO-A. The results presented herein could be applied in the rational structure-based design and optimization of β-carbolines towards preclinical candidates that would target the MAO-A enzyme and would be applicable especially in the treatment of mental disorders such as depression.

## 1. Introduction

Monoamine oxidases (MAOs) are an important group of enzymes responsible for the oxidative deamination of amine neurotransmitters, primarily in the central nervous system, thereby regulating their levels. When the function of MAOs is increased, this can lead to the development of mental disorders (e.g., autism and depression), and even their normal function is associated with the production of hydrogen peroxide, which can lead to neuronal damage and the development of neurodegenerative disorders (Parkinson’s or Alzheimer’s disease).

MAOs exist in two distinct isoforms, MAO-A and MAO-B, which share about 70% homology in their sequence but differ in some parts of their structure, tissue distribution, and selectivity—MAO-A preferentially metabolizes serotonin and norepinephrine, whereas MAO-B predominantly degrades dopamine [1]. Both isoenzymes contain in their active site the FAD (flavin adenine dinucleotide) co-factor bound to one of the cysteine residues. The overall catalytic cycle of MAOs begins with the conversion of the amine substrate to the corresponding imine, which is followed by the regeneration of the reduced flavin cofactor (FADH_2_) with molecular oxygen. The latter is the part of the catalytic cycle in which hydrogen peroxide is formed. The rate-limiting step of this cycle is the cleavage of the C-H bond, vicinal to the amino group and the subsequent transfer of the hydrogen atom to the flavin cofactor. There are several proposals for the mechanism of the rate-limiting step [2,3,4,5,6,7,8], with most evidence pointing to the hydride transfer mechanism [9,10,11,12,13].

Because of their involvement in the development of neurodegenerative and neuropsychiatric diseases, MAOs are intriguing targets for drug development, several of which are already on the market. The best-known drugs are the MAO-B-specific rasagiline and selegiline (both used mainly to treat Parkinson’s disease) and the MAO-A-specific moclobemide (used mainly to treat depression). In addition, studies have shown that smoking can also affect the activity of MAO. Results interestingly suggest that tobacco smokers have up to 28% lower brain MAO-A activity and 40% lower brain MAO-B activity compared with nonsmokers [14,15]. Considering the involvement of MAOs in the development of neurodegenerative diseases, this may be related to the lowest rate of Parkinson’s disease in smokers [16]. Several studies suggest that β-carbolines which are found primarily in tobacco smoke, coffee, dark chocolate, salmon, raisins, certain spices, cooked foods, and alcoholic beverages, among others [17], are responsible for the inhibition of MAO [18].

**Figure 1:**
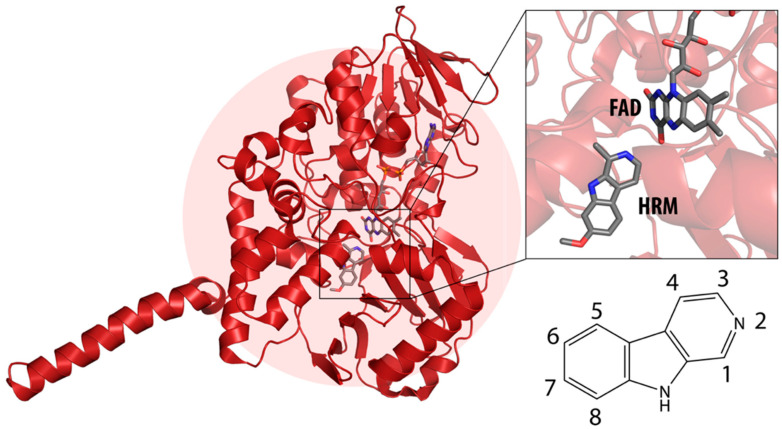
**Left**: monoamine oxidase A enzyme. **Top right**: Active site of monoamine oxidase A with the flavin adenine nucleotide (FAD) cofactor and the bound β-carboline harmine (HRM). **Bottom right**: basic tricyclic structural element and the numbering of the β-carbolines.

β-carbolines (or harmala alkaloids) are naturally occurring, biologically active alkaloids that are derivatives of indole [19] and whose basic structural element is shown in Figure 1. They were first isolated from the plant *Peganum harmala*, a succulent in the *Nitrariaceae* family [19], but are also found in several other plant families [20]. Some of the harmala alkaloids are the reason for the hallucinogenic properties of *ayahuasca*, a psychoactive drink used in spiritual ceremonies in South America and Africa [21,22]. They not only inhibit MAO enzymes but are also known to produce a variety of different effects in the body, with their mechanism of action being associated with imidazole [23,24], serotonin [25], benzodiazepine [26] and dopamine receptors [27,28], as well as affecting cerebral neurotransmitter concentrations [29]. They have also been shown to intercalate into DNA [30,31,32], interact with DNA topoisomerases [31], and have a protective role against oxidative stress in human tissues when accumulated [33]. Other studies focused on their antiplatelet [34], antimalarial [35] and antidepressant effects [36]. The latter, in particular, was the main topic of many studies and some specific β-carbolines have been analyzed in detail. Harman has been shown to reduce depressive behavior in animals, presumably through several different modes of action [37,38,39], the most important of which is MAO-A inhibition [40]. Similarly, preclinical studies have shown that harmine has a potential antidepressant effect in various depression models [39,41,42]. Currently WHO estimates that depression affects about 280 million people worldwide. This gives rise to the urgent need to develop new antidepressants that can effectively treat depression and improve the quality of life of those affected [43].

We can divide the β-carbolines into three groups based on the saturation of one of their rings: fully aromatic β-carbolines, dihydro-β-carbolines which are partially saturated, and fully saturated tetrahydro-β-carbolines [44]. All usually contain different substituents on one of their three rings. Depending on the surrounding solvent and pH, the compounds can exist in one of four states: as a cation, in neutral form, as a zwitterion, or as an anion [45]. Studies focusing on the selectivity of some β-carbolines have found that a methyl group on the C1 atom appears to be critical for the compounds to be selective towards MAO-A rather than MAO-B—norharman (without substituents) is 10–20 times less active as a MAO-A inhibitor, but two orders of magnitude more active as a MAO-B inhibitor than harman or harmaline (both have a methyl group at position 1) [46]. A crystal structure of one of the fully aromatic β-carbolines (harmine, methyl group at position 1 and a methoxy group at position 7) bound to the active site of MAO-A was determined [47] (Figure 1) and harmine was characterized as a reversible MAO-A inhibitor. This crystal structure presents an excellent starting point for our computational studies.

The aim of the present work was to study the binding properties and molecular recognition between β-carbolines and the MAO-A isoform at the atomistic level by applying a variety of computational approaches. The study is based on a series of differently substituted β-carbolines [48], and we sought to first gain a static insight into their binding and later extended this with a more dynamic approach. Within the latter, we performed molecular dynamics (MD) simulations of the considered β-carbolines and analyzed their geometry (RMSD), interactions with the MAO-A active site (dynophores) and calculated their predicted binding free energy (with the linear interaction energy method). All the data obtained could be used to develop new MAO-A selective inhibitors that could potentially be used in the effective treatment of mental disorders, especially depression.

## 2. Results

### 2.1. Static Insight into the β-Carboline–MAO-A Molecular Recognition

We began our computational analysis of a selected series of β-carbolines **1–11**, which bind non-covalently into the active site of the MAO-A isoform, with a static view of our system. A crystal structure of MAO-A with harmine (compound **6**), a β-carboline that acts as a reversible MAO-A inhibitor, had already been determined (PDB code 2Z5X), providing an excellent starting point for our study [47]. Thus, we were able to first redock harmine into the MAO-A active site and deduce whether our docking software is able to reproduce the observed X-ray binding pose. For this purpose, we used three different scoring functions, available in GOLD [49], namely: GoldScore, ChemScore and ChemPLP. All three scoring functions were able to successfully reproduce the X-ray binding pose (see Supplementary Materials, Figure S14). Consequently, we were able to proceed with docking the remainder of the compounds studied.

**Figure 2:**
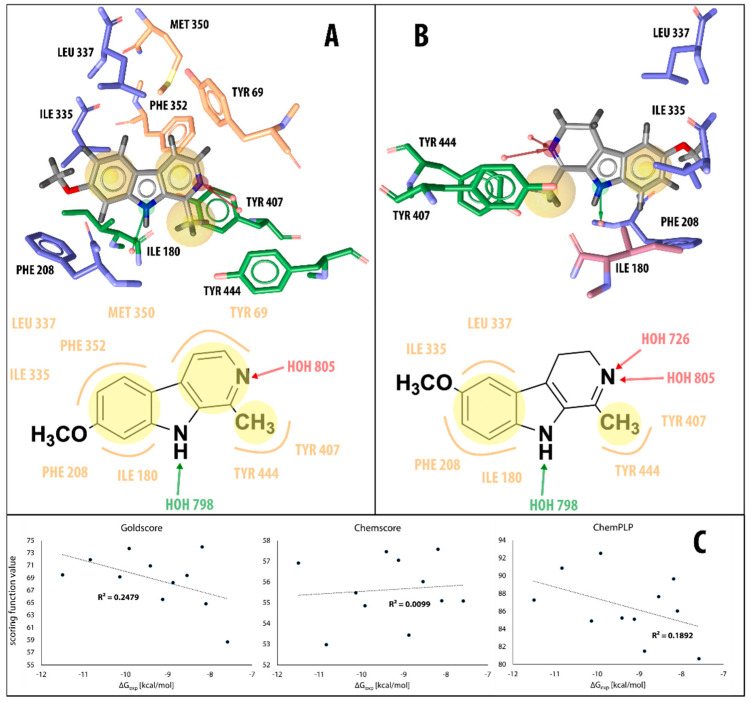
(**A**) 3D (top) and 2D (bottom) structure-based pharmacophore model for harmine (compound **6**) docked in the MAO-A active site. (**B**)) 3D (top) and 2D (bottom) structure-based pharmacophore model for 6-methoxy harmalan (compound **10**) docked in the MAO-A active site. Yellow spheres represent favorable areas of the ligand for hydrophobic interactions while green and red arrows denote the identified H-bond donors and acceptor interactions, respectively. (**C**) GoldScore, ChemScore and ChemPLP scoring functions compared to experimentally observed binding free energy of compounds **1–11**.

Using the LigandScout program package [50], we then visualized the key intermolecular interactions by generating a 3D structure-based pharmacophore for harmine (compound **6**, Figure 2A), 6-methoxyharmalan (compound **10**, Figure 2B), and other β-carbolines based on the binding modes determined using the GoldScore scoring function. In the X-ray-determined binding model of harmine in the MAO-A active site, researchers previously concluded that harmine interacts with Tyr69, Asn181, Phe208, Val210, Gln215, Cys323, Ile325, Ile335, Leu337, Phe352, Tyr407, Tyr444, and the cofactor flavin adenine dinucleotide (FAD), with seven water molecules occupying the space between harmine and these amino acids [47]. Our pharmacophore model detects interactions of harmine with six of these groups (Tyr69, Phe208, Phe352, Ile335, Tyr407, and Tyr444) as well as an additional interaction with Ile180 and hydrogen bonds with the conserved water molecules (Figure 2A).

We can roughly divide the studied set of β-carbolines into two substructural groups: those that are neutral (compounds **1**, **3**, **6**, **9**, and **10**) and those that have a positive charge on the pyridine nitrogen atom (**2**, **4**, **5**, **7**, **8**, and **11**), with some important differences in the pharmacophores of the two groups. All neutral compounds (except inhibitor **9**) exhibit hydrophobic interactions of all three aromatic β-carboline rings with MAO-A active site residues, namely tyrosine (Tyr407), isoleucines (Ile180, Ile335), leucine (Leu337), phenylalanines (Phe208, Phe352), and methionine (Met350). The addition of a methyl group to the inhibitor structure leads to a further hydrophobic interaction with the active site tyrosines (Tyr407 and Tyr444). In all neutral β-carbolines, the pyridine nitrogen atom acts as a hydrogen bond acceptor (interaction with tyrosine residues), while the pyrrole nitrogen acts as a hydrogen bond donor (interaction with the conserved water molecules). The pharmacophore models predict no additional interactions as a result of the addition of the methoxy group (compounds **6**, **9**, and **10**).

For the β-carbolines with a positive charge on the pyridine nitrogen atom (compounds **2**, **4**, **5**, **7**, **8**, and **11**), pharmacophore models generally predict fewer interactions than for the neutral β-carbolines. The focus in this group is on the hydrophobic interactions formed between the active site residues and the benzene ring portion of the β-carboline structure. In contrast to what we found for the neutral β-carboline group, in this case, we see interactions occurring with the oxygen atom of the methoxy group. The latter acts as a hydrogen bond acceptor with water or with one of the MAO-A active site tyrosines. In addition, for compounds **8** and **11**, the model predicts an interaction between the positively charged nitrogen atom and a phenylalanine residue.

Furthermore, we wanted to determine if there was a correlation between the experimentally determined binding free energies of the β-carbolines and the values predicted by all three scoring functions available in GOLD: GoldScore, ChemScore, and ChemPLP. That is, we wanted to determine whether a more negative free energy of binding (i.e., a smaller inhibition constant *K_i_*, meaning that the compound is a better inhibitor) corresponds to a higher scoring function value and vice versa. Our results show that there is little to no correlation between the values of the scoring functions and the experimentally determined binding free energy (see Figure 2C). This is not surprising since docking is usually a good predictor of the binding geometry but generally fails in exactly predicting the binding energy [51]. To obtain this information, we need to use more thorough dynamical methods [52], so we have extended our study to include MD simulations.

### 2.2. Binding Free Energies of β-Carbolines

**Figure 3:**
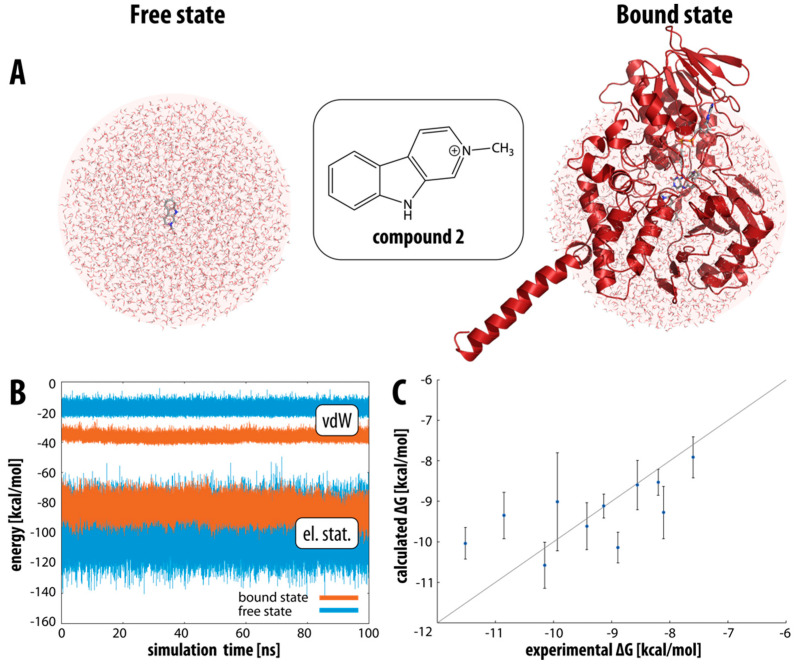
(**A**) Outline of the defined bound and free state for compound **2**. (**B**) Interaction energy decomposition for compound **2**: van der Waals (top, vdW) and electrostatic (bottom, el. stat.) energy component between compound **2** and its surroundings—water in the free state (blue) and solvated protein in the bound state (orange) during the whole molecular dynamics simulation. (**C**) Experimental- and LIE-calculated binding free energies together with error bars for compounds **1–11**.

Following the established linear interaction energy (LIE) protocol, we first calculated the interaction energies between compounds **1–11** in the aqueous environment (free state, see Figure 3A, left) and in the protein active site (bound state, see Figure 3A, right). For all compounds considered, the interaction energy in the bound state is more favorable than in the free state (by about 3–10 kcal mol^−1^, depending on the compound), which is in line with our expectations and indicates that the binding of the compounds is energetically favored. Interestingly, splitting the interaction energy into the van der Waals and electrostatic components shows that the bound state is energetically more favorable with respect to the van der Waals component, while the opposite is true for the electrostatic component. This is not surprising since the active site of MAO-A is highly hydrophobic (as confirmed by our additional detailed active site analysis), so it is to be expected that van der Waals interactions are the predominant driving force for binding these β-carbolines. The compounds may form more electrostatic interactions in water than in the very hydrophobic active site. 

In general, the observed fluctuations (i.e., standard deviations) for the electrostatic contributions of charged compounds **1**, **3**, **6**, **9**, and **10** (average standard deviation of 1.9 kcal mol^−1^) are higher than for neutral compounds **2**, **4**, **5**, **7**, **8**, and **11** (average standard deviation of 1.0 kcal mol^−1^), which is consistent with our expectations because the electrostatics of charged moieties are expected to fluctuate more than those of their neutral counterparts. The fluctuations for the van der Waals contributions are generally smaller, with no significant difference between the charged and uncharged β-carbolines (0.7 and 0.6 kcal mol^−1^, respectively) (Table 1).

Figure 3B shows the values of the van der Waals and electrostatic components of the interaction energy for β-carboline **2** over the course of the MD simulation in the bound and free states. The van der Waals component of the interaction energy varies between −10 and −25 kcal mol^−1^ in the free state and between −30 and −40 kcal mol^−1^ in the bound state. There is a clear separation between the two states, with a clear preference for the bound state. On the other hand, the electrostatic component varies between −70 and −100 kcal mol^−1^ in the bound state and between −70 and −130 kcal mol^−1^ in the free state, with a clear overlap between the two states. In this case, the fluctuations are slightly larger than for the van der Waals component, and we can infer a slight preference for the free state, but the distinction between the two states is much less pronounced.

The experimentally determined inhibition constants (*K_i_*) for the examined compounds were taken from ref. [48]. The binding free energy was calculated from these values by using the standard formula:

(1) $$∆G_{binding}=RT\ln K_{i}$$

where *R* is the gas constant and *T* is the temperature. The binding free energy was calculated according to the LIE formalism, as described in the computational methods section. The values determined for the coefficients α, β and γ were 0.54, 0.26 and −2.65, respectively, resulting in the final LIE equation:

(2) $$∆G_{binding}=0.54\left(\langle V_{L-P}^{vdW}\rangle -\langle V_{L-W}^{vdW}\rangle \right)+0.26\left(\langle V_{L-P}^{el}\rangle -\langle V_{L-W}^{el}\rangle \right)-2.65$$

Coefficient α is, therefore, parameterized to be larger than coefficient β, giving greater weight to the van der Waals component of the interaction energy, which in turn is consistent with the hydrophobicity of the active site and the compounds themselves. It should be noted that similar α and β coefficients have been frequently determined in LIE studies of various bio-macromolecular systems [53,54,55]. The experimentally derived and the calculated binding free energies for all investigated β-carbolines are listed in Table 2, along with the average RMSD values of the ligand for the duration of the MD simulation.

The binding free energy values calculated from our MD simulations are in broad agreement with the available experimental data. Figure 3C further illustrates the correlation between the experimentally determined and the calculated free energies of binding together with the standard deviations. Looking at the deviations, most of the values fall on the diagonal line, which is a perfect match between the experimental and calculated free energies, with some inhibitors deviating to a larger extent such as compounds **2**, **6**, **8**, and **10**.

### 2.3. Dynamic Insight into the β-Carboline–MAO-A Molecular Recognition

The obtained MD trajectories of all ligands **1–11** in the bound state provide a considerable amount of structural data for further analysis to allow a more dynamic picture of the molecular recognition between β-carbolines and the active site of the MAO-A enzyme. To complement the binding free energy data obtained by LIE calculations, we investigated the interaction between β-carbolines and MAO-A using dynamic pharmacophore models. The dynamic pharmacophore (dynophore) approach allows us to combine the features of MD simulations and static 3D structure-based pharmacophore models, as it provides us with the statistical characterization of the pharmacophore features of the ligand throughout the simulation. This information is then distilled into so-called “superfeatures” represented by the clouds of a given pharmacophore model [56,57,58]. Furthermore, the dynamic picture of molecular recognition was complemented by RMSD calculations to evaluate the stability of the proposed ligand-binding pose.

**Figure 4:**
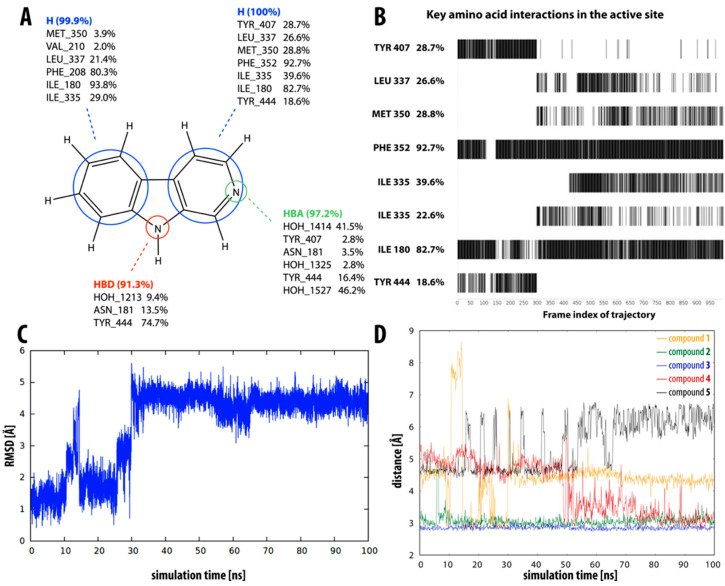
Overview of the interaction pattern obtained through dynophore analysis for β-carboline compound **1**. (**A**) Calculated percentage of the occurrence of a dynamic pharmacophore element based on all frames divided into element-interacting amino acid pairs (for compounds **2–5,** see Supplementary Materials, Figure S12). (**B**) The presence of hydrophobic interactions of the pyridine ring with various active site amino acid residues during the molecular dynamics simulation. (**C**) RMSD of compound **1** during the molecular dynamics simulation (for compounds **2–5**, see Supplementary Materials, Figures S2–S5). (**D**) Distance between the pyrrole nitrogen and residue Asn181 during the molecular dynamics simulation for compounds **1–5**. The distance is shown as a block average over 1 ns (1000 total blocks of 0.1 ns cover the entire 100 ns MD simulation).

Compound **1** (i.e., norharman) is the simplest of the β-carbolines studied, having no additional methyl or methoxy groups. It is also the least potent inhibitor, as indicated by the experimentally determined inhibition constant (*K_i_* = 3.34 ± 0.10 µM), which is converted to a binding free energy value of −7.6 kcal mol^−1^. This was well reflected in our LIE calculations, with compound **1** having the lowest calculated binding free energy of −7.9 ± 0.5 kcal mol^−1^. This observation could be related to the highest RMSD value in the group of simulated β-carbolines, i.e., compound **1** undergoes a significant geometric shift at about the 30 ns mark of the simulation (see Figure 4C). Before the shift, this compound is seen in a binding pose similar to the X-ray-determined pose of β-carboline harmine (compound **6**), whereas after the shift, it occupies a different part of the active site. It no longer interacts with the aromatic cage tyrosines (Tyr407 and Tyr444), which have been shown to be very important for binding [59]. This pose is, therefore, not so well-suited for MAO-A inhibition, which is clearly reflected in our results, especially in the dynophore analysis (Figure 4A,B). The latter shows hydrophobic interactions between the aromatic rings of inhibitor **1** and several active site amino acids (Ile180, Ile335, Leu337, Met350, Phe352, Tyr407, and Tyr444) as well as the pyridine and pyrrole nitrogen atoms, which act as hydrogen bond acceptor and donor, respectively. Frame-by-frame analysis clearly shows that the interactions with both tyrosines are present in the first 30 ns of the simulation. After the molecule moves away from the aromatic cage, new interactions appear with Leu337, Met350, and Ile335.

β-carbolines **2–5** represent variations in compound **1** with additional methyl groups at different positions. In all compounds, most of the interactions are hydrophobic and originate mainly from the two aromatic rings. In compounds **2**, **4**, and **5**, there is an additional methyl group on the pyridine nitrogen, which makes it positively charged and unable to act as a hydrogen bond acceptor, as is the case in compounds **1** and **3**. An additional methyl group on the C1 atom (next to the pyridine nitrogen, see Figure 1) in compounds **3**, **4,** and **5** is able to form additional stabilizing hydrophobic interactions with the active site tyrosines (Tyr197, Tyr407, andTyr444) and isoleucines (Ile180 and Ile207). Interestingly, in compound **3**, the pyrrole nitrogen can act as a hydrogen bond donor and interact with Asn181.

Figure 4D shows the distance between the pyrrole nitrogen and Asn181 for compounds **1–5** during the MD simulation. For inhibitors **2** and **3**, this distance is relatively stable, with the average distance for compound **3** being slightly shorter, presumably resulting in the interaction being reflected in the dynophore analysis. For the other three inhibitors, the distance is less stable and for inhibitor **1**, we can see a clear correlation between the distance and the interaction pattern in the dynophore analysis. Namely, the dynophore analysis reflects the interaction between Asn181 and the pyrrole nitrogen when the distance is shorter than about 2.9 Å.

All four compounds in this group have relatively small RMSD values (see Table 2), implying that their binding modes are stable throughout the MD simulation. Moreover, the calculated LIE values for the free energy of binding of inhibitors **3–5** agree remarkably well with the experimentally determined values for their free energies of binding.

**Figure 5:**
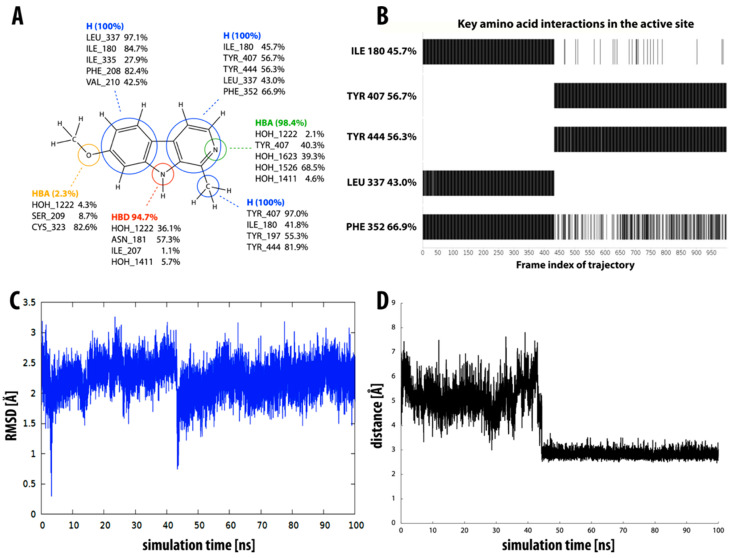
Overview of the interaction pattern obtained through dynophore analysis for β-carboline harmine **6**. (**A**) Calculated percentage of the occurrence of a dynamic pharmacophore element based on all frames divided into element-interacting amino acids pairs (for compounds **7–9**, see Supplementary Materials, Figure S13). (**B**) The presence of hydrophobic interactions of the pyridine ring with various active site amino acid residues during the molecular dynamics simulation. (**C**) RMSD of compound **6** during the molecular dynamics simulation (for all compounds, see Supplementary Materials, Figures S1–S11). (**D**) Distance between the pyrrole nitrogen and Asn181 during the molecular dynamics simulation.

According to experimental data, β-carboline **6** (i.e., harmine) is the strongest MAO-A inhibitor in the series (*K_i_* = 0.005 ± 0.0002 µM), and the binding free energy value we calculated also places it among the strongest inhibitors (Δ*G_calc_* = −10.0 ± 0.4). Structurally, it is most similar to inhibitor **3**, with an additional methoxy group present at the C7 atom. Therefore, the predicted interactions (see Figure 5A) are similar to those of compound **3**: hydrophobic interactions of the two aromatic rings and the methyl group, and the pyridine and pyrrole nitrogen as hydrogen bond acceptor and donor, respectively. Additional interactions are possible with the methoxy group.

Dynophore analysis predicts interactions with Leu337, Ile180, Ile335, Phe208, Val210, Tyr407, Tyr444, Phe352, Ser209, Cys323, Tyr197, Asn181, and Ile207. This covers 9 of the 12 interactions determined in the X-ray structure (compared to 6 of 12 that we determined using static pharmacophore analysis). The RMSD for compound **6** is relatively large, and (similar to inhibitor **1**) we can see a clear shift in the molecule at about the 40 ns mark of the simulation (Figure 5C). In contrast to compound **1**, the molecule moves closer to the aromatic tyrosine cage. Figure 5B clearly shows the appearance of interactions with Tyr407 and Tyr444 at this time point and the disappearance (or weakening) of interactions with Leu337, Ile180, and Phe352. At the same time point, we also note an abrupt shortening of the distance between the pyrrole nitrogen and residue Asn181 and a subsequent stabilization of this distance (Figure 5D).

Similar to inhibitor **6**, compounds **7–9** also have a methoxy group at the C7 atom, with additional methyl groups at various positions. The dynophore analysis predicts hydrophobic interactions of aromatic rings and the methyl group at the C1 atom, as well as additional interactions of the methoxy oxygen atom with Cys323 and water molecules. Analogously to compound **6**, some interactions with the pyrrole or pyridine nitrogen atom can also be formed. The calculated RMSD values for inhibitors **7–9** are relatively small, while the agreement between the experimentally determined free energies of binding and their calculated counterparts is not optimal.

**Figure 6:**
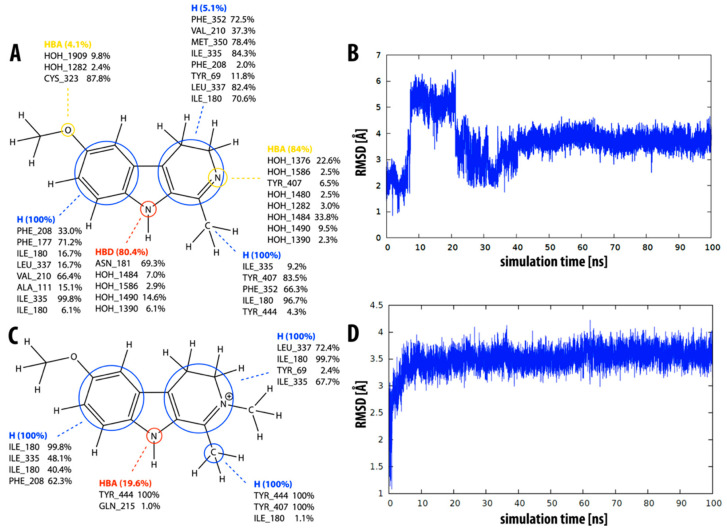
Overview of the interaction pattern obtained by dynophore analysis for compounds **10** (top) and **11** (bottom). Calculated percentage of the occurrence of a dynamic pharmacophore element based on all frames divided into element-interacting amino acids pairs for compound **10** (**A**) and compound **11** (**C**). Calculated RMSD value for the duration of the molecular dynamics simulation for compound **10** (**B**) and compound **11** (**D**).

Finally, compounds **10** and **11** both have a methoxy group on the C6 atom instead of the C7 atom. The dynophore analysis of both inhibitors (Figure 6A,C) again shows mainly the presence of hydrophobic interactions with both rings and the methyl group at C1. The prevalence of hydrophobic interactions with the pyridine ring is significantly lower for inhibitor **10**, probably due to there being one less double bond. The average calculated RMSD value is quite high for both inhibitors (3.7 Å and 3.5 Å, respectively). Compound **10** clearly moves away from the aromatic cage at 10–20 ns of the MD simulation (Figure 6B). On the other hand, compound **11** undergoes a larger geometric shift in the first 5 ns of the simulation and remains relatively stable thereafter (Figure 6D). The calculated binding free energies for compounds **10** and **11** are −10.1 and −8.5 kcal mol^−1^, respectively, the latter being in better agreement with the experimentally determined values than the former.

### 2.4. Guidelines for Further Optimization of β-Carbolines as Non-Covalent MAO-A Inhibitors

The low molecular weight of the investigated β-carboline compounds, as well as several substitution possibilities provided by their tricyclic core scaffold, suggests that these compounds could be further optimized to obtain even more active and pharmacokinetically more suitable preclinical candidates targeting the MAO-A enzyme. Furthermore, these β-carbolines can be classified as natural products which are known to be the most productive source of lead compounds to develop new drug molecules [60,61].

**Figure 7:**
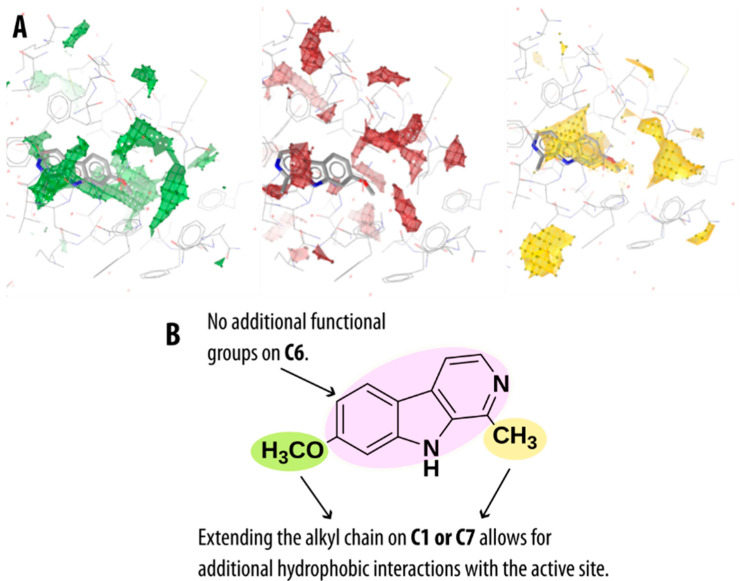
(**A**) Calculated molecular interaction fields in the MAO-A active site using different molecular probes. Regions of the active site with amino acids capable of acting as hydrogen bond donors (green), acceptors (red) or forming hydrophobic interactions (yellow). (**B**) General β-carboline structure with proposed possibilities for rational structure-based optimization of MAO-A inhibitory activity.

Previous docking studies of MAO-A and MAO-B enzymes with β-carboline derivatives have shown that the addition of lipophilic and bulky groups at C7 increases the inhibitory potency against MAO-A more than against MAO-B enzyme [62]. This is due to the structurally unfavorable overlap of these derivatives as well as some of the β-carbolines in this study substituted at the C7 position with Tyr326 residue of MAO-B, as compared to the structurally smaller Ile335 found in the MAO-A isoform [47]. The mentioned residues comprise one of the crucial differences between the two MAO isoforms and play an important role in determining the compound’s binding selectivity. The advantage of the β-carbolines substituted at C7 was further substantiated by our analysis of the MAO-A active site using molecular interaction fields (MIFs). The contours of the obtained MIF using a hydrophobic probe identified an empty lipophilic pocket and the possibility of hydrophobic interactions with residues Ile180, Phe208, Ile325, and Ile335. The other amino acid residues identified by our Apo Site Grid analysis (see Figure 7A) as capable of forming hydrophobic interactions were Tyr69, Ile207, Leu337, Met350, Phe352, and the aromatic cage tyrosines Tyr407 and Tyr444. For all these residues, our pharmacophore and dynophore analysis also revealed favorable hydrophobic interactions.

In addition, MIFs further revealed some MAO-A active site residues as favorable to form hydrogen bonds, namely Asn181, Gln215, Ser209, and Thr336—all of which, except for the latter, we also found to form interactions with the pyrrole or pyridine nitrogen using the dynophore analysis. However, since the active site is predominantly hydrophobic, hydrogen bonding is possible mainly with the water molecules present in the MAO-A binding cavity (for the average number of water molecules present in the binding site cavity during the MD simulations, see Supplementary Materials, Table S12). Based on all the collected data from the Apo Site Grid analysis as well as the previous structure and energy data from the MD simulations, we present some basic ideas for optimizing the β-carboline structure to fully utilize all the possible interactions within the MAO-A active site (see Figure 7B). The main possible points of optimization are carbon atoms C1 and C7, where the alkyl chain could potentially be extended to allow for more hydrophobic interactions with the active site. The C6 atom should be left unsubstituted, since the addition of functional groups on this atom does not contribute positively to the MAO-A inhibitory activity.

## 3. Discussion

With this computational study, we aimed to provide additional insight into MAO-A molecular recognition by examining the binding modes of a series of substituted β-carboline natural products. MAOs are enzymes with a variety of important functions in the central nervous system, so the development of drugs targeting these enzymes could be useful for several therapeutic applications. For instance, they show potential for development of novel drugs for treatment of neurodegeneration, while drugs targeting the MAO-A isoform are particularly useful in the treatment of mental disorders such as depression.

After docking a set of 11 β-carbolines into the MAO-A active site, the derived structure-based pharmacophores revealed key interactions with most of the amino acids identified as important in the X-ray-determined binding model of harmine **6**, with an emphasis on the occurrence of hydrophobic interactions with the active site. There were minor differences in the binding modes of the neutral β-carbolines compared to their charged counterparts, with the charged β-carbolines exhibiting fewer interactions. While docking is invaluable for evaluating the geometric aspects of β-carboline binding, it is less adequate for predicting binding energies. Thus, we used the linear interaction energy (LIE) approach and generated MD simulations for all inhibitors as well as explored the molecular recognition feature with dynamic pharmacophores (i.e., dynophores). Similar to the obtained pharmacophores, the generated dynophores predicted mainly hydrophobic interactions with the active site residues along with some hydrogen bonding of the pyrrole and pyridine nitrogen with the water molecules present in the active site and with some amino acid residues. In addition, during the performed MD simulations, we also examined the positional changes in the MAO-A active site residues compared to their original positions in the X-ray structure. We found that no significant shifts in the most important residues (e.g., Tyr69, Ile180, Phe208, Leu337, Phe352, Tyr407, and Tyr444) occurred, suggesting that no substantial reorganization of the MAO-A active site is required for the non-covalent binding of β-carbolines.

Decomposition of the interaction energy into electrostatic and van der Waals contributions within the LIE formalism showed that van der Waals interactions appear to be the predominant driving force for β-carboline binding, consistent with the observed high hydrophobicity of the MAO-A binding site. The empirical LIE coefficients α, β, and γ were determined to be 0.54, 0.26, and −2.65, respectively, and the calculated free energies of binding were in good agreement with the experimentally determined values.

Dynamic pharmacophore analysis confirms that the relative orientation of the β-carbolines is important for their ability to form interactions with the targeted active site. Most importantly, stabilizing interactions with the tyrosines of the so-called aromatic cage (Tyr407 and Tyr444) are possible only when the β-carboline is in a parallel position such as the conformation obtained by X-ray crystallography. Other sites that appear to be important for the formation of interactions are the C1 atom, where the methyl group at this position can form hydrophobic interactions, and the C7 atom, where the methoxy group at this position forms additional hydrogen bonds with active site water molecules and various amino acid residues such as Ser209 and Cys323.

The results presented in this study thus provide a static and dynamic atomistic picture of molecular recognition occurring between substituted β-carbolines and MAO-A. Furthermore, the data obtained can serve as guidelines in further rational optimization and development of β-carbolines towards active compounds that would potentially be useful as MAO-A-specific inhibitors with therapeutic application in the treatment of depression.

## 4. Materials and Methods

### 4.1. Molecular Docking and Binding Site Analysis

Molecular docking studies were performed using the available co-crystal structure of the β-carboline harmine (compound **6**) with the MAO-A enzyme isoform (PDB code 2Z5X) and the GOLD (v. 5.3.0) docking program [49]. The binding site included all atoms within a radius of 20 Å around the center, which was fixed to the N5 atom of the FAD moiety. Prior to docking, all water molecules were removed except for seven water molecules occupying the binding cavity, which were identified as important for the binding modes and were therefore retained, and all were used in the docking procedure [47]. The ligand obtained from the crystal structure (harmine, i.e., compound **6**) was set as the reference ligand for the docking procedure and the conformations of the remaining ligands were generated as described in the next subsection. Standard protonation states of amino acid residues at neutral pH were determined. A set of 25 algorithm runs were performed for each ligand. To achieve optimal accuracy in the docking process, the following genetic algorithm settings were used: population size 100, selection pressure 1.1, number of operations 100,000, 5 islands, and niche size 2. The scoring functions used to evaluate the ligand positions obtained from the docking studies were GoldScore, ChemScore and ChemPLP. Docking results were visualized in LigandScout [50], which was also used to determine the 3D structure-based pharmacophores of the binding modes considered.

LigandScout was also used for the Apo Site Grid analysis with the default settings used. During this task, molecular probes, such as hydrogen bond acceptor, hydrogen bond donor, positive ionizable, negative ionizable, hydrophobic probes, and others, scanned the β-carboline binding site of the MAO-A enzyme, deriving contours of the corresponding molecular interaction fields (MIFs).

### 4.2. Preparation of the Initial Structures for Molecular Dynamics Simulations

Our MD studies were also all based on the experimentally solved crystal structure of the MAO-A enzyme with the bound reversible inhibitor harmine (compound **6**) obtained from the Protein Data Bank (PDB code 2Z5X). The studied structures of β-carbolines **1–11** were prepared manually by modifying the experimentally determined compound **6** with PyMOL [63]. To obtain structures **9–11**, the corresponding pyridine bond had to be hydrogenated, resulting in a partially unsaturated ring. Molecules **2**, **4**, **7**, and **11** were modified by methylation of the pyridine ring nitrogen and molecules **5** and **8** by additional methylation of the pyrrole ring nitrogen resulting in six charged β-carbolines. The methoxy group of compound **6** had to be removed to give compounds **1–5**. In addition, deletion of the methyl group yielded compound **1**.

Before starting the MD simulations, the force field parameters had to be developed for all the compounds we studied. The simulations were performed using the OPLS-AA force field [64]. After all the compounds were extracted from their initial complexes, they were fully optimized at the Hartree-Fock (HF) level of theory using the 6–31G(d) basis set encoded in the Gaussian16 program package [65]. For all compounds, atomic charges were determined by fitting to the HF/6–31G(d) calculated electrostatic potential according to the RESP scheme as implemented in AmberTools18 [66]. Parameters were determined using the ffld_server utility assisted by the Maestro v. 11.7 graphical interface [67]. The MAO-A enzyme was parametrized following the procedure described in our previous work [68]. Parameters and atom types for all simulated ligands **1–11** are available in the Supporting Information (see Supplementary Materials, Tables S1–S11).

All crystal waters were removed from the original protein complex and a spherical cell of TIP3P waters with a radius of 30 Å centered on the crystal coordinates of the central N5 atom was constructed. Similarly, for the free-state simulations, a spherical cell of TIP3P waters with the same radius of 30 Å centered on the N5 atom of the ligands was prepared. For the positively charged ligands, a chloride ion was included in both the bound and free-state simulations to ensure electroneutrality. All topology and coordinate files needed to initiate the MD simulations were created using the qprep5 utility of the MD package Q v. 5.06 [69].

### 4.3. Molecular Dynamics Simulations

The calculation of binding free energies for our compounds was facilitated by generating MD trajectories of the studied compounds in the bound and free states using the qdyn5 utility of the MD package Q v. 5.06 [69].

In the free state, a position constraint of 100 kcal mol^−1^ Å^−2^ was imposed on the central nitrogen atom of all studied compounds to prevent diffusion of the compound to the edge of the simulation sphere. In addition, a flat-bottom harmonic potential of 75 kcal mol^−1^ Å^−2^ was placed on the chloride ion for the charged compounds in both states and set to zero for distances less than 26.5 Å from the center of the simulation sphere.

Protein atoms beyond the 30 Å spherical cell were restrained to their original crystal structure coordinates using harmonic constraints. Non-bonding interactions of these atoms were turned off. The local reaction field (LRF) method was used to evaluate long-range electrostatics for distances beyond the 10 Å cut-off. For distances below 10 Å, the non-bonding interactions were explicitly evaluated.

All systems were first relaxed in 14 separate steps, by slowly increasing the temperature and the step size. Initially, two separate simulations were performed with 30,000 and 20,000 steps at 5 K and a step size of 0.005 fs and 0.01 fs, respectively. For the bound state, all protein and ligand atoms were constrained to their original coordinates, allowing only the movement of water molecules. Then, the system was further relaxed in four additional stages of 20,000 steps and one of 50,000 steps, all at 5 K, with step sizes of 0.01 fs, 0.04 fs, 0.1 fs, 0.3 fs, and 1.0 fs, respectively. Then, the system was heated in five stages of 40,000 steps with a step size of 1.0 fs in 50 K increments starting at 50 K and ending at 250 K. Finally, two simulations of 10,0000 steps were performed at 298 K, with a step size of 1.0 and 2.0 fs, respectively. Data for the calculation of binding free energies were collected from 10 consecutive simulations with 5,000,000 steps and a step size of 2.0 fs at 298 K (NVT canonical ensemble), giving a total of 0.1 μs MD simulation time for each investigated compound in both the free and bound states.

Visualization and analysis of the generated MD trajectories were facilitated by the VMD program package [70]. RMSD analysis was performed for the entire trajectory, and the calculated RMSD values were calculated referring to the initial structure of the protein and ligand, respectively. All calculations were performed using the computational facilities of the Ažman High-Performance Computing Center of the National Institute of Chemistry.

### 4.4. Generation of Dynamical Pharmacophore Models

To obtain a more detailed description of our system in terms of dynamic pharmacophore (dynophore) models, 1000 exported MD equidistant frames were used in dynamic pharmacophore analysis with the DynophoreApp developed at the Molecular Design Lab of Freie Universität (FU) Berlin [58]. These dynophore calculations were performed on computers at the Molecular Design Lab, Berlin, Germany, and subsequently visualized in LigandScout [50].

### 4.5. Calculation of Binding Free Energies Using the Linear Interaction Energy Method

**Figure 8:**
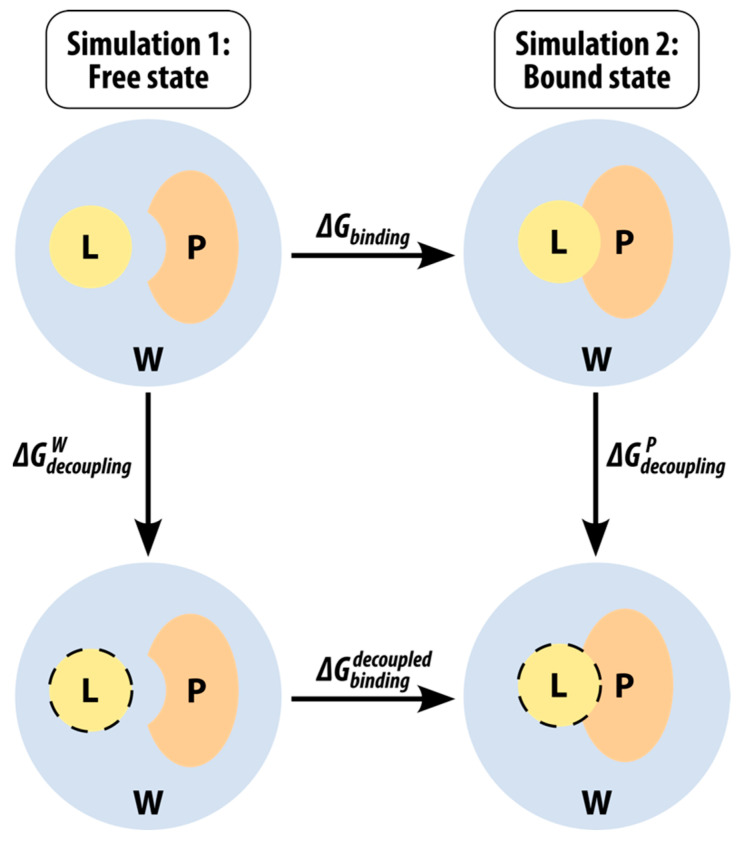
Thermodynamic cycle used in LIE binding free energy calculations. *P* stands for protein, *L* for ligand and *W* for water. The dashed line around the ligand represents decoupling (turning the non-bonding electrostatic and van der Waals interactions off).

The linear interaction energy (LIE) method [63,71] was developed by Åqvist to facilitate the calculation of binding free energies of ligands that bind to their target in a non-covalent way (*G_bind_*). We have successfully used it in our previous study, where we calculated the binding free energies of N-sulphonyl-glutamic acid inhibitors of MurD ligase [72]. LIE is based on a modified linear response approximation for electrostatic interactions and on an empirical term treating the non-polar interactions. The main advantage of LIE compared to other methods that can be used for the same purpose (e.g., free energy perturbation and thermodynamic integration) is that we only need to perform two MD simulations—one in the free state (i.e., solvated, in water) and one in the bound state (i.e., solvated protein). This means that the sampling of any intermediate states is not necessary which consequently drastically reduces the associated computational costs. A thermodynamic cycle can be drawn as outlined in Figure 8, where the top row represents the two physical states mentioned above and the bottom row represents two unphysical intermediate states.

An equation can be derived because the free energy is a state function and, therefore, the sum of the changes in the thermodynamic cycle must be zero:

(3) $$∆G_{binding}+∆G_{decoupling}^{P}-∆G_{decoupling}^{W}-∆G_{binding}^{decoupled}=0.$$

Since, for a decoupled ligand, all non-bonding interactions are switched off, the $∆G_{binding}^{decoupled}$ term is zero, and the equation can be rearranged as:

(4) $$∆G_{binding}=∆G_{decoupling}^{W}-∆G_{decoupling}^{P}.$$

The two terms on the right-hand side of the equation essentially represent the difference in free energy for the transfer of the ligand from its environment (*W* represents water or the free state, while *P* represents the protein or the bound state) to the gas phase. In the context of LIE, these can be written as:

(5) $$∆G_{solvation}^{Q}=-∆G_{decoupling}^{Q}=\alpha V_{L-Q}^{vdW}+\beta V_{L-Q}^{el}+\gamma ,$$

where the 〈⬚〉 brackets denote the MD mean values of the non-bonded van der Waals (*vdW*) and electrostatic (*el*) interactions between the ligand *L* and its environment *Q* (i.e., either the solvated protein binding site *P* or the aqueous solution *W*). Consequently, the final LIE equation has the following form:

(6) $$∆G_{binding}=\alpha \left(\langle V_{L-P}^{vdW}\rangle -\langle V_{L-W}^{vdW}\rangle \right)+\beta \left(\langle V_{L-P}^{el}\rangle -\langle V_{L-W}^{el}\rangle \right)+\gamma .$$

The empirical parameters of this equation are the coefficients α and β for the nonpolar and polar binding energy contributions, respectively, and an additional (optional) constant γ, which is used mainly when we are dealing with very hydrophobic active sites [73]. In our study, we determined the empirical coefficients α, β, and γ for our system using the linear approximation method based on the van der Waals and electrostatic energy averages in the bound and free states during the MD simulation.

## Figures and Tables

**Table 1:** Average van der Waals and electrostatic interaction energy between β-carbolines **1–11** (*L*) and their surroundings (solvated protein *P* or aqueous solution *W*) obtained from MD simulations. Standard deviations are also given and were calculated from ten subsequent molecular dynamics simulation steps.

Compound	$\begin{array}{c} \langle V_{L-P}^{vdW}\rangle \\ \left[\mathrm{kcal}\;{\mathrm{mol}}^{-1}\right] \end{array}$	$\begin{array}{c} \langle V_{L-W}^{vdW}\rangle \\ \left[\mathrm{kcal}\;{\mathrm{mol}}^{-1}\right] \end{array}$	$\begin{array}{c} \langle V_{L-P}^{vdW}-V_{L-W}^{vdW}\rangle \\ \left[\mathrm{kcal}\;{\mathrm{mol}}^{-1}\right] \end{array}$	$\begin{array}{c} \langle V_{L-P}^{el}\rangle \\ \left[\mathrm{kcal}\;{\mathrm{mol}}^{-1}\right] \end{array}$	$\begin{array}{c} \langle V_{L-W}^{el}\rangle \\ \left[\mathrm{kcal}\;{\mathrm{mol}}^{-1}\right] \end{array}$	$\begin{array}{c} \langle V_{L-P}^{el}-V_{L-W}^{el}\rangle \\ \left[\mathrm{kcal}\;{\mathrm{mol}}^{-1}\right] \end{array}$
**1**	−28.4 ± 0.5	−15.4 ± 0.1	−13.1 ± 0.5	−15.1 ± 1.6	−22.2 ± 0.1	+7.1 ± 1.6
2 *	−36.7 ± 0.5	−17.9 ± 0.1	−18.9 ± 0.5	−84.1 ± 2.2	−98.0 ± 0.2	+13.9 ± 2.2
**3**	−32.5 ± 0.3	−16.8 ± 0.1	−15.7 ± 0.3	−15.7 ± 0.9	−23.6 ± 0.1	+7.9 ± 0.9
4 *	−38.2 ± 0.8	−19.4 ± 0.1	−17.7 ± 0.8	−82.9 ± 1.6	−96.9 ± 0.1	+14.0 ± 1.6
5 *	−39.8 ± 1.0	−22.7 ± 0.1	−17.1 ± 1.0	−77.6 ± 0.7	−86.5 ± 0.2	+8.9 ± 0.7
**6**	−36.9 ± 0.4	−19.6 ± 0.1	−17.2 ± 0.4	−17.0 ± 1.2	−24.5 ± 0.1	+7.2 ± 1.2
7 *	−42.3 ± 1.0	−22.0 ± 0.1	−20.3 ± 1.0	−78.9 ± 4.1	−96.7 ± 0.2	+17.8 ± 4.1
8 *	−43.9 ± 0.7	−25.3 ± 0.1	−18.6 ± 0.7	−74.5 ± 1.6	−87.5 ± 0.2	+12.9 ± 1.6
**9**	−41.9 ± 1.0	−20.6 ± 0.1	−21.2 ± 1.0	−9.7 ± 0.6	−23.4 ± 0.1	+13.7 ± 0.6
**10**	−39.5 ± 0.6	−20.7 ± 0.1	−18.8 ± 0.6	−12.9 ± 0.7	−23.3 ± 0.1	+10.4 ± 0.7
11 *	−41.0 ± 0.2	−22.6 ± 0.1	−18.4 ± 0.2	−81.1 ± 1.1	−96.8 ± 0.2	+15.7 ± 1.1

* Inhibitors that are charged.

**Table 2:** Calculated binding free energies of β-carbolines **1–11** compared with the available experimental results (obtained from ref. [48]) and average RMSD values for each ligand (for RMSD graphs of all compounds along the duration of molecular dynamics simulations see Supplementary Materials, Figures S1–S11).

Compound	Structure	$\begin{array}{c} K_{i}\\ \left[µM\right] \end{array}$	$\begin{array}{c} ∆G_{binding}^{exp}\\ \left[\mathrm{kcal}\;{\mathrm{mol}}^{-1}\right] \end{array}$	$\begin{array}{c} ∆G_{binding}^{calc}\\ \left[\mathrm{kcal}\;{\mathrm{mol}}^{-1}\right] \end{array}$	RMSD [Å]
**1**	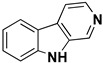	3.34 ± 0.10	−7.6 ± 0.02	−7.9 ± 0.5	3.7
**2**	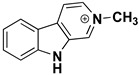	1.43 ± 0.09	−8.1 ± 0.04	−9.3 ± 0.6	1.1
**3**	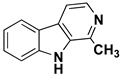	0.26 ± 0.024	−9.1 ± 0.06	−9.1 ± 0.3	1.9
**4**	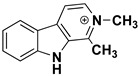	0.68 ± 0.026	−8.6 ± 0.02	−8.6 ± 0.6	1.5
**5**	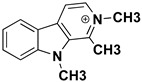	0.16 ± 0.036	−9.4 ± 0.14	−9.6 ± 0.6	1.6
**6**	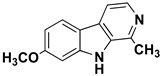	0.005 ± 0.0002	−11.5 ± 0.02	−10.0 ± 0.4	2.3
**7**	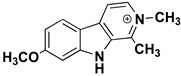	0.069 ± 0.008	−9.9 ± 0.07	−9.0 ± 1.2	1.7
**8**	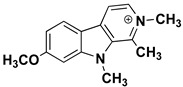	0.015 ± 0.0008	−10.9 ± 0.03	−9.4 ± 0.6	1.4
**9**	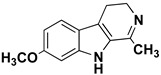	0.048 ± 0.007	−10.2 ± 0.09	−10.6 ± 0.6	1.9
**10**	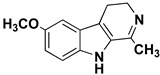	0.39 ± 0.052	−8.9 ± 0.08	−10.1 ± 0.4	3.7
**11**	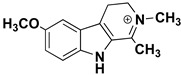	1.23 ± 0.27	−8.2 ± 0.01	−8.5 ± 0.3	3.5

## Data Availability

Not applicable.

## Supplementary Materials

The following supporting information can be downloaded at: https://www.mdpi.com/article/10.3390/molecules27196711/s1, Tables S1–S11: Parameters and atom types for compounds 1–11; Figures S1–S11: RMSD graphs for compounds 1–11; Figure S12: Overview of the interaction pattern obtained with dynophore analysis for compounds 2–5; Figure S13: Overview of the interaction pattern obtained with dynophore analysis for compounds 7–9; Figure S14: Comparison between X-ray binding pose of harmine with the determined binding modes; Table S12: The average number of water molecules present in the MAO-A active site during the simulation.

## Sample Availability

Samples of the compounds are not available from the authors.
