# Supplementary material for: Computational Insights into β-Carboline Inhibition of Monoamine Oxidase A
Source: Molecules. 2022 Oct 9;27(19):6711. doi: 10.3390/molecules27196711 (PMC9571839; doi:10.3390/molecules27196711)
Supplement: Supplementary file 1 [file molecules-27-06711-s001.zip › molecules-1947102-supplementary.pdf]

Supporting Information

Computational Insights into β-Carboline Inhibition of Monoamine Oxidase A

Alja Prah,[1,2] Tanja Gavranić,[1,3] Andrej Perdih,[1,3] Marija Sollner Dolenc,[3] Janez Mavri[,1]*

[1] Theory Department, National Institute of Chemistry, Slovenia

[2] Networking Infrastructure Centre, Jožef Stefan Institute, Slovenia

[3] University of Ljubljana, Faculty of Pharmacy, Slovenia

** Correspondence: janez.mavri@ki.si*

Table S1. Parameters and atom types for compound 1.

Name	Atom type	Charge	Name	Atom type	Charge
**N1**	NB	-0.627167	**C10**	CA	-0.258372
**C1**	CA	0.277849	**C11**	CA	-0.044640
**N2**	NA	-0.659257	**H1**	HA	0.074191
**C2**	CA	0.346683	**H2**	HA	0.138254
**C3**	CA	-0.524251	**H3**	HA	0.155958
**C4**	C56B	0.314435	**H4**	HA	0.160158
**C5**	C56B	-0.004619	**H5**	HA	0.181165
**C6**	C56B	0.433887	**H6**	HA	0.058143
**C7**	CA	-0.375403	**H7**	HA	0.206540
**C8**	C56B	-0.165691	**H8**	H	0.421296
**C9**	CA	-0.109160			

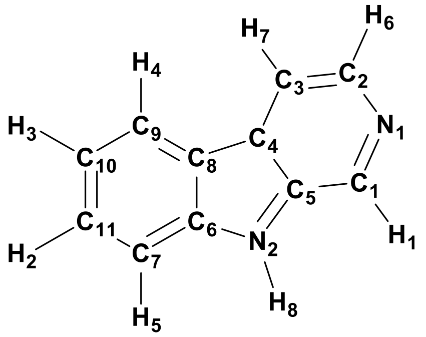

Table S2. Parameters and atom types for compound 2.

Name	Atom type	Charge	Name	Atom type	Charge
**C1**	CA	-0.124700	**C12**	CT	-0.185016
**N1**	NAP	0.034460	**H1**	HC	0.154979
**C2**	CA	-0.070145	**H2**	HC	0.154979
**N2**	NA	-0.624408	**H3**	HC	0.154979
**C3**	CA	-0.248609	**H4**	HA	0.181944
**C4**	C56B	0.146988	**H5**	HA	0.173293
**C5**	C56B	0.198083	**H6**	HA	0.161804
**C6**	C56B	0.397271	**H7**	HA	0.200980
**C7**	CA	-0.355814	**H8**	HA	0.200813
**C8**	C56B	-0.067840	**H9**	HA	0.215975
**C9**	CA	-0.148086	**H10**	HA	0.214076
**C10**	CA	-0.201283	**H11**	H	0.440122
**C11**	CA	-0.004845			

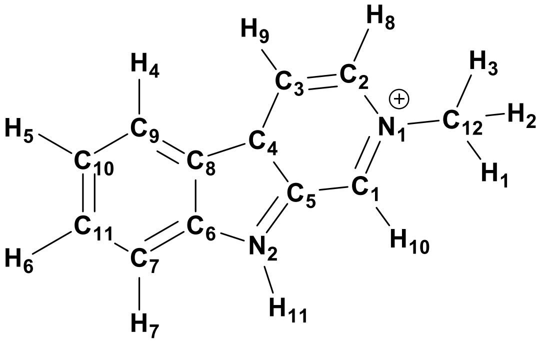

Table S3. Parameters and atom types for compound 3.

Name	Atom type	Charge	Name	Atom type	Charge
**C1**	CT	-0.523721	**C11**	CA	-0.233281
**N1**	NB	-0.658430	**C12**	CA	-0.065902
**C2**	CA	0.595521	**H1**	HA	0.177082
**N2**	NA	-0.541782	**H2**	HA	0.136823
**C3**	CA	0.240226	**H3**	HA	0.164965
**C4**	CA	-0.435624	**H4**	HA	0.174390
**C5**	C56B	0.176056	**H5**	HA	0.097254
**C6**	C56B	-0.090646	**H6**	HA	0.179458
**C7**	C56B	0.373985	**H7**	HC	0.139919
**C8**	CA	-0.375332	**H8**	HC	0.139919
**C9**	C56B	-0.029445	**H9**	HC	0.139919
**C10**	CA	-0.177414	**H10**	H	0.396061

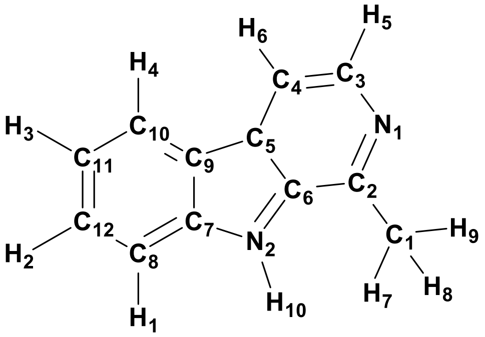

Table S4. Parameters and atom types for compound 4.

Name	Atom type	Charge	Name	Atom type	Charge
**C1**	CA	0.314960	**C13**	CT	-0.369953
**N1**	NAP	-0.005213	**H1**	HA	0.190157
**C2**	CA	-0.130805	**H2**	HA	0.181759
**N2**	NA	-0.509171	**H3**	HA	0.197921
**C3**	CA	-0.244798	**H4**	HA	0.159727
**C4**	C56B	0.078153	**H5**	HC	0.144194
**C5**	C56B	-0.030148	**H6**	HA	0.211289
**C6**	C56B	0.360353	**H7**	HA	0.226637
**C7**	CA	-0.374663	**H8**	HC	0.144194
**C8**	C56B	0.019461	**H9**	HC	0.144194
**C9**	CA	-0.190045	**H10**	HC	0.146419
**C10**	CA	-0.188390	**H11**	HC	0.146419
**C11**	CA	-0.012521	**H12**	HC	0.146419
**C12**	CT	-0.164595	**H13**	H	0.408045

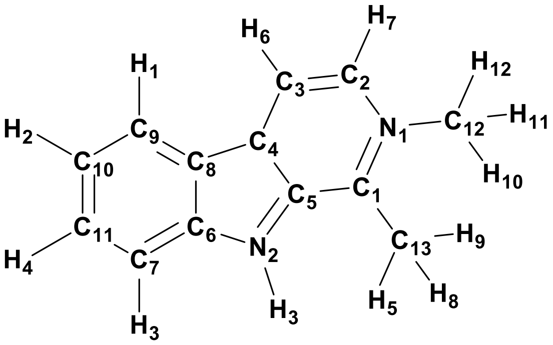

Table S5. Parameters and atom types for compound 5.

Name	Atom type	Charge	Name	Atom type	Charge
**C1**	CA	0.293121	**H1**	HA	0.166798
**N1**	NAP	-0.023254	**H2**	HA	0.180803
**C2**	CA	-0.133842	**H3**	HA	0.158483
**N2**	NA	-0.056208	**H4**	HA	0.183084
**C3**	CA	-0.195988	**H5**	HC	0.146197
**C4**	C56B	0.069653	**H6**	HA	0.214972
**C5**	C56B	-0.077079	**H7**	HA	0.203382
**C6**	C56B	0.181622	**H8**	HC	0.120573
**C7**	CA	-0.291303	**H9**	HC	0.120573
**C8**	C56B	0.025576	**H10**	HC	0.120573
**C9**	CA	-0.172491	**H11**	HC	0.146197
**C10**	CA	-0.179275	**H12**	HC	0.146197
**C11**	CA	-0.027107	**H13**	HC	0.146986
**C12**	CT	-0.163345	**H14**	HC	0.146986
**C13**	CT	-0.195627	**H15**	HC	0.146986
**C14**	CT	-0.403242			

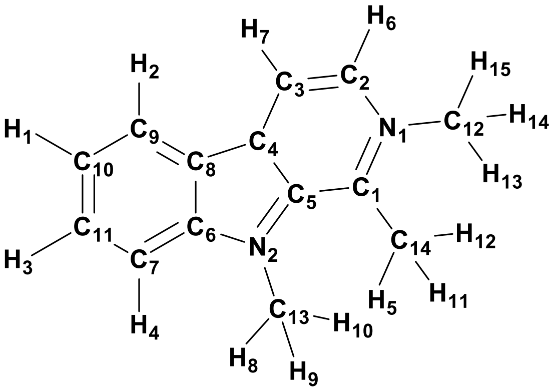

Table S6. Parameters and atom types for compound 6.

Name	Atom type	Charge	Name	Atom type	Charge
**C1**	CT	-0.357709	**C12**	CA	0.548725
**N1**	NB	-0.681270	**C13**	CT	0.027979
**O1**	OS	-0.383457	**H1**	HA	0.080821
**C2**	CA	0.621389	**H2**	HA	0.204471
**N2**	NA	-0.455290	**H3**	HA	0.192071
**C3**	CA	0.294448	**H4**	HA	0.161627
**C4**	CA	-0.536078	**H5**	HA	0.194987
**C5**	C56B	0.363125	**H6**	HC	0.093373
**C6**	C56B	-0.225204	**H7**	HC	0.093373
**C7**	C56B	0.312346	**H8**	HC	0.093373
**C8**	CA	-0.498308	**H9**	H	0.372831
**C9**	C56B	-0.210456	**H10**	HC	0.062240
**C10**	CA	-0.024785	**H11**	HC	0.062240
**C11**	CA	-0.469101	**H12**	HC	0.062240

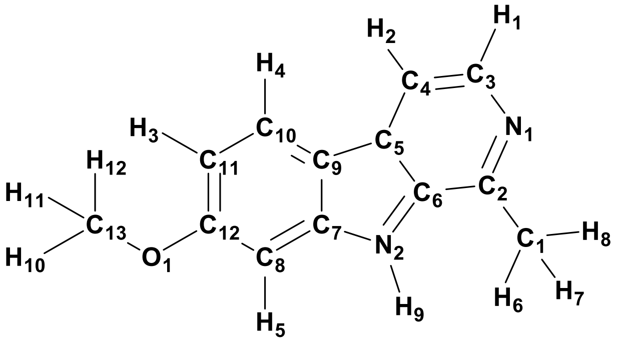

Table S7. Parameters and atom types for compound 7.

Name	Atom type	Charge	Name	Atom type	Charge
**C1**	CA	0.297264	**H1**	HC	0.144399
**N1**	NAP	-0.035333	**H2**	HC	0.144399
**C2**	CA	-0.134899	**H3**	HC	0.144399
**N2**	NA	-0.508775	**H4**	HC	0.083733
**C3**	CA	-0.145909	**H5**	HC	0.083733
**C4**	C56B	0.100178	**H6**	HC	0.083733
**C5**	C56B	-0.051447	**H7**	HA	0.186213
**C6**	C56B	0.454071	**H8**	HA	0.199258
**C7**	CA	-0.646777	**H9**	HA	0.247531
**C8**	C56B	-0.160466	**H10**	HA	0.218259
**C9**	CA	-0.131442	**H11**	HA	0.216955
**C10**	CA	-0.358781	**O1**	OS	-0.369729
**C11**	CA	0.628524	**H12**	HC	0.151850
**C12**	CT	-0.181445	**H13**	HC	0.151850
**C13**	CT	-0.381808	**H14**	HC	0.151850
**C14**	CT	0.002102	**H15**	H	0.416511

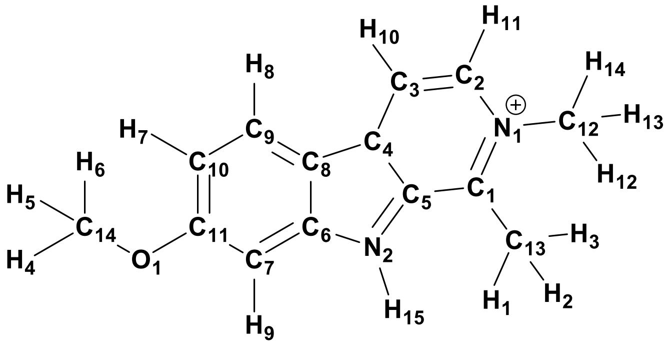

Table S8. Parameters and atom types for compound 8.

Name	Atom type	Charge	Name	Atom type	Charge
**C1**	CA	0.274315	**H2**	HC	0.035193
**N1**	NAP	0.091401	**H3**	HC	0.035193
**C2**	CA	-0.139962	**H4**	HA	0.244872
**N2**	NA	0.077014	**H5**	HA	0.195140
**C3**	CA	-0.235150	**H6**	HA	0.217880
**C4**	C56B	0.115165	**H7**	HA	0.226170
**C5**	C56B	-0.143271	**H8**	HA	0.203693
**C6**	C56B	0.186189	**H9**	HC	0.143710
**C7**	CA	-0.486059	**H10**	HC	0.149475
**C8**	C56B	-0.032649	**H11**	HC	0.149475
**C9**	CA	-0.153367	**H12**	HC	0.149475
**C10**	CA	-0.353112	**H13**	HC	0.143710
**C11**	CA	0.529660	**H14**	HC	0.143710
**C12**	CT	-0.286091	**O1**	OS	-0.402910
**C13**	CT	0.128488	**H15**	HC	0.150805
**C14**	CT	-0.307855	**H16**	HC	0.150805
**C15**	CT	-0.387110	**H17**	HC	0.150805
**H1**	HC	0.035193			

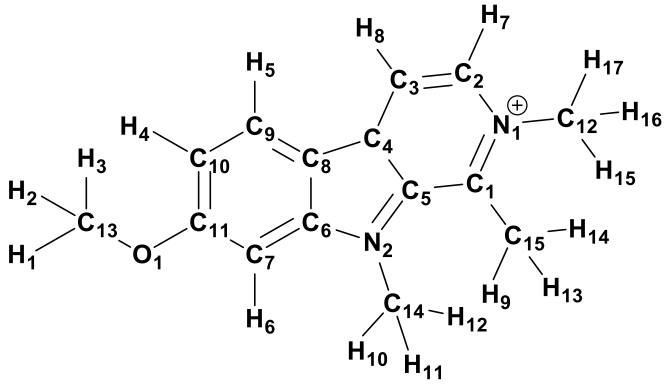

Table S9. Parameters and atom types for compound 9.

Name	Atom type	Charge	Name	Atom type	Charge
**C1**	CT	-0.474064	**C13**	CT	0.481459
**N1**	NI	-0.730741	**H1**	HA	0.163041
**O1**	OS	-0.387008	**H2**	HA	0.190115
**C2**	CD	0.695351	**H3**	HA	0.197476
**N2**	NA	-0.442773	**H4**	HA	-0.044034
**C3**	C5B	0.116397	**H5**	HA	0.090443
**C4**	C5A	-0.214237	**H6**	HA	0.090443
**C5**	C56A	0.288777	**H7**	HA	-0.044034
**C6**	CA	-0.501025	**H8**	HA	0.121525
**C7**	C56B	-0.153271	**H9**	HA	0.121525
**C8**	CA	-0.048741	**H10**	HA	0.121525
**C9**	CA	-0.461798	**H11**	H	0.370836
**C10**	CA	0.541308	**H12**	HC	0.056286
**C11**	CT	0.043539	**H13**	HC	0.056286
**C12**	CT	-0.300895	**H14**	HC	0.056286

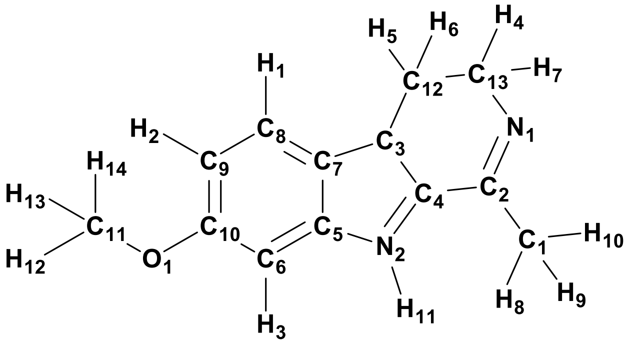

Table S10. Parameters and atom types for compound 10.

Name	Atom type	Charge	Name	Atom type	Charge
**C1**	CT	-0.456900	**H1**	HA	0.194599
**N1**	NI	-0.710167	**H2**	HC	0.047052
**C2**	CD	0.671601	**H3**	HA	0.168913
**N2**	NA	-0.458449	**H4**	HA	0.181209
**C3**	CT	0.445969	**H5**	HC	0.047052
**C4**	CT	-0.294499	**H6**	HC	0.047052
**C5**	C5B	0.087881	**H7**	HC	0.087832
**C6**	C5A	-0.156832	**H8**	HC	0.087832
**C7**	C56A	0.169326	**H9**	HC	-0.034114
**C8**	CA	-0.186125	**H10**	HC	-0.034114
**C9**	C56B	-0.042593	**O1**	OS	-0.391438
**C10**	CA	-0.356187	**H11**	HC	0.118760
**C11**	CA	0.427084	**H12**	HC	0.118760
**C12**	CA	-0.338720	**H13**	HC	0.118760
**C13**	CT	0.070257	**H14**	H	0.370199

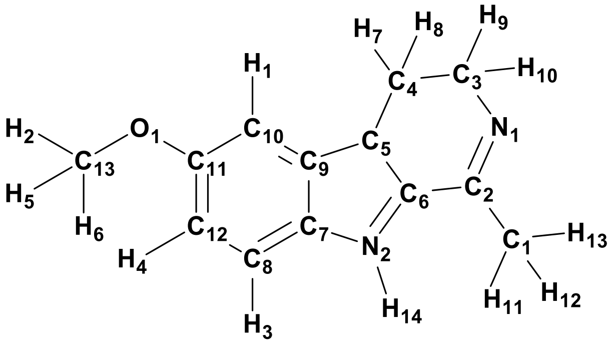

Table S11. Parameters and atom types for compound 11.

Name	Atom type	Charge	Name	Atom type	Charge
**C1**	CM	0.406343	**H2**	HC	0.072194
**N1**	NE	-0.183108	**H3**	HC	0.072194
**C2**	CT	-0.027202	**H4**	HC	0.123986
**N2**	NIP	-0.431395	**H5**	HC	0.123986
**C3**	CT	-0.187076	**H6**	HC	0.123986
**C4**	CM	0.119921	**H7**	HC	0.190101
**C5**	CM	-0.174237	**H8**	HC	0.191122
**C6**	CG	0.216533	**H9**	HC	0.188536
**C7**	CM	-0.230202	**H10**	HC	0.101415
**C8**	CM	-0.007605	**H11**	HC	0.101415
**C9**	CM	-0.381159	**H12**	HC	0.124900
**C10**	CM	0.446756	**H13**	HC	0.124900
**C11**	CM	-0.218144	**O1**	OS	-0.375086
**C12**	CT	-0.138609	**H14**	HC	0.136566
**C13**	CT	0.031355	**H15**	HC	0.136566
**C14**	CT	-0.281383	**H16**	HC	0.136566
**H1**	HC	0.072194	**H17**	H	0.393672

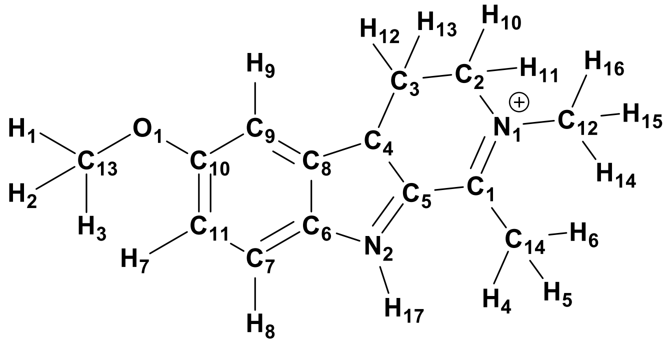

Figure S1. RMSD graph for compound 1.

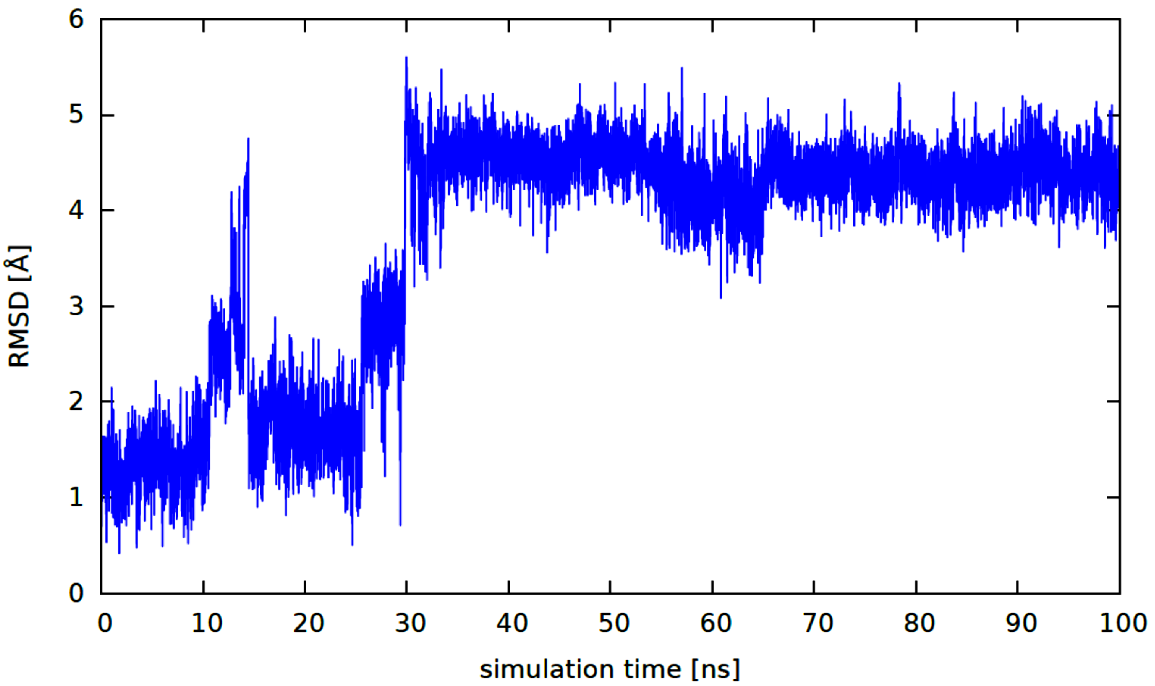

Figure S2. RMSD graph for compound 2.

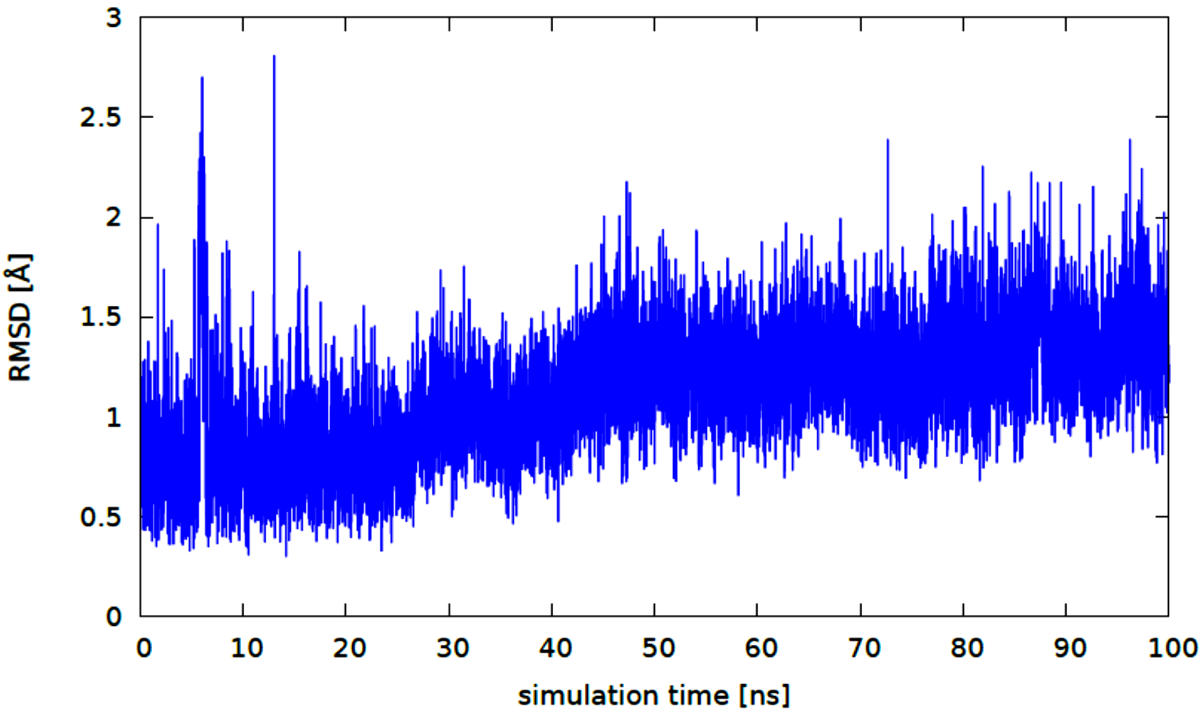

Figure S3. RMSD graph for compound 3.

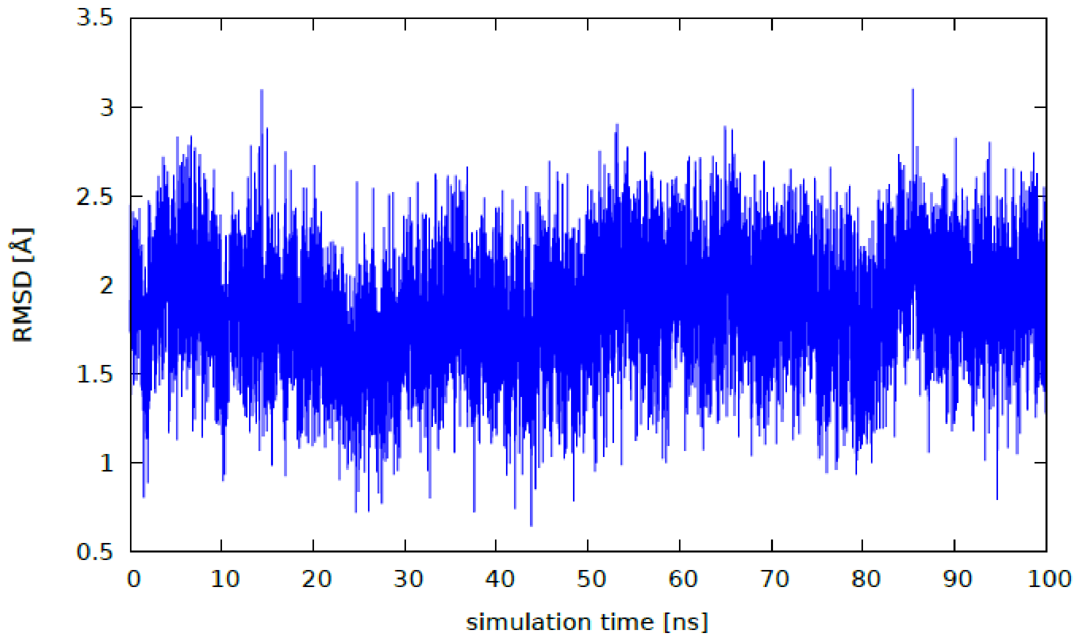

Figure S4. RMSD graph for compound 4.

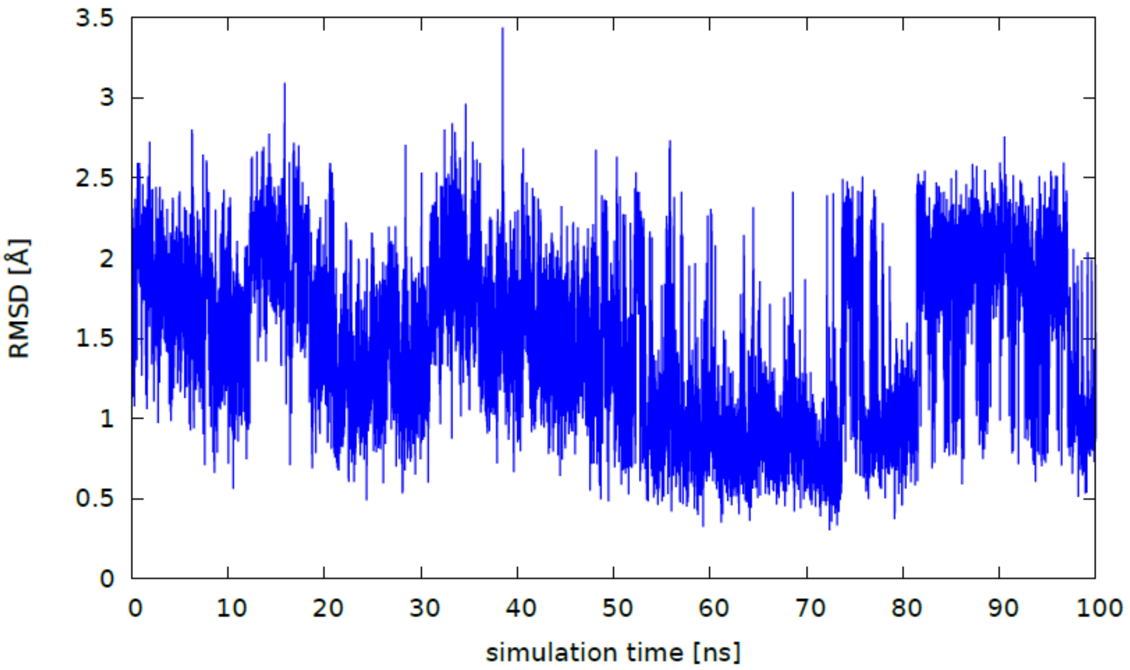

Figure S5. RMSD graph for compound 5.

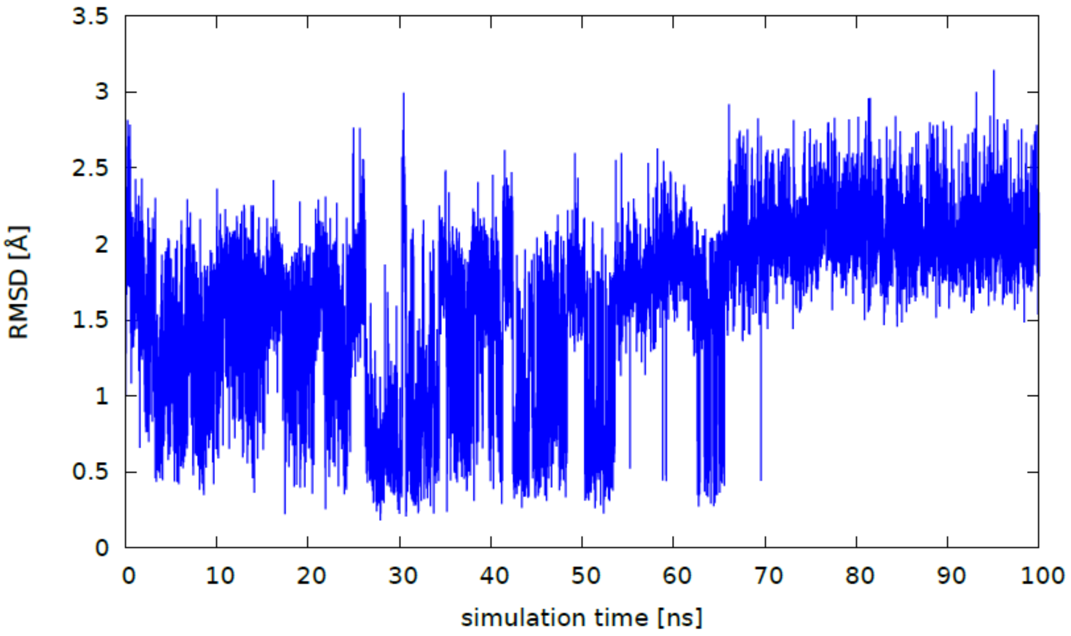

Figure S6. RMSD graph for compound 6.

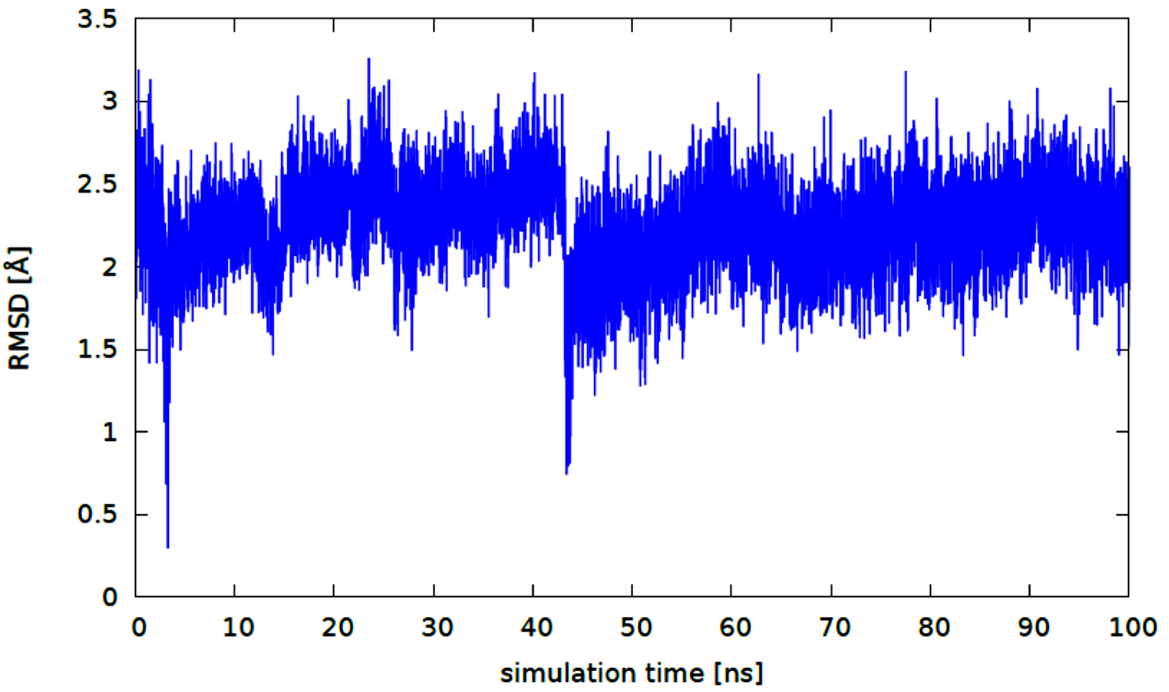

Figure S7. RMSD graph for compound 7.

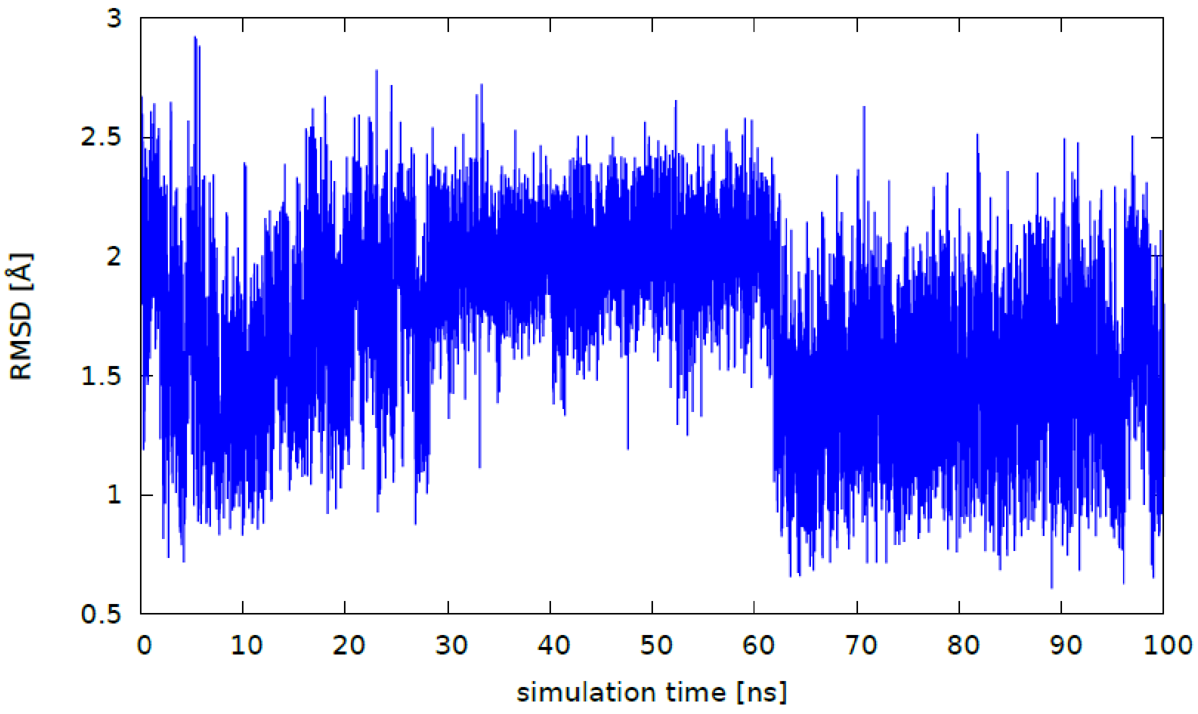

Figure S8. RMSD graph for compound 8.

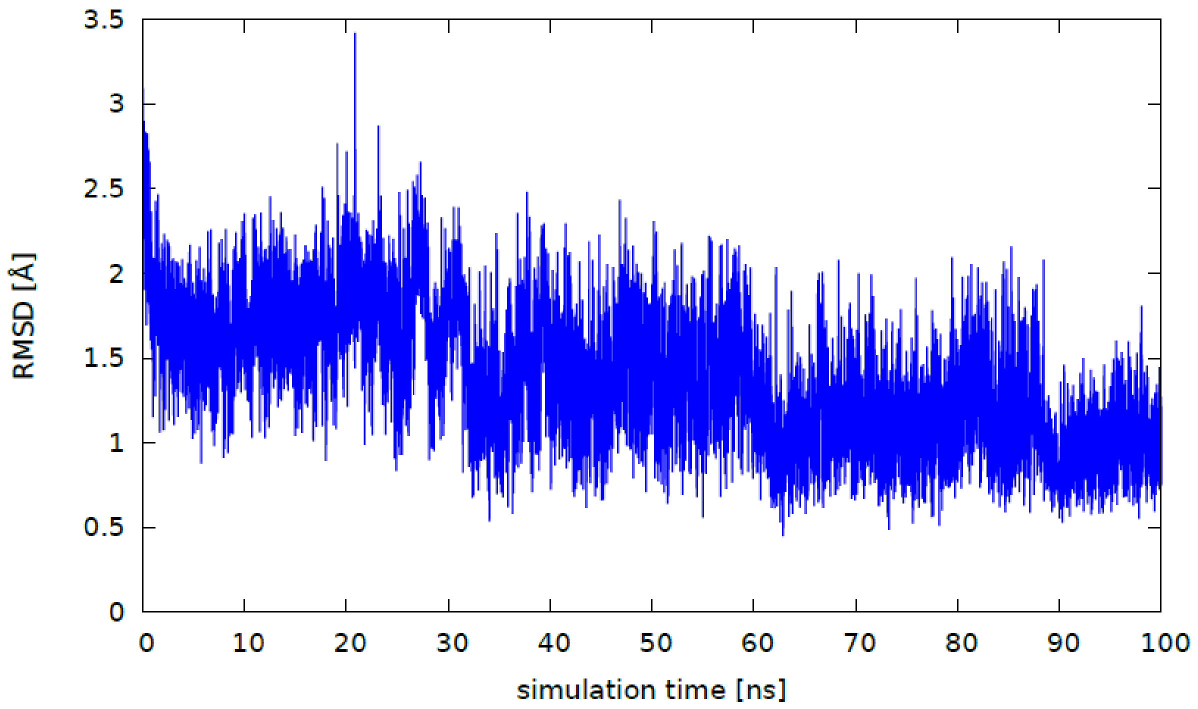

Figure S9. RMSD graph for compound 9.

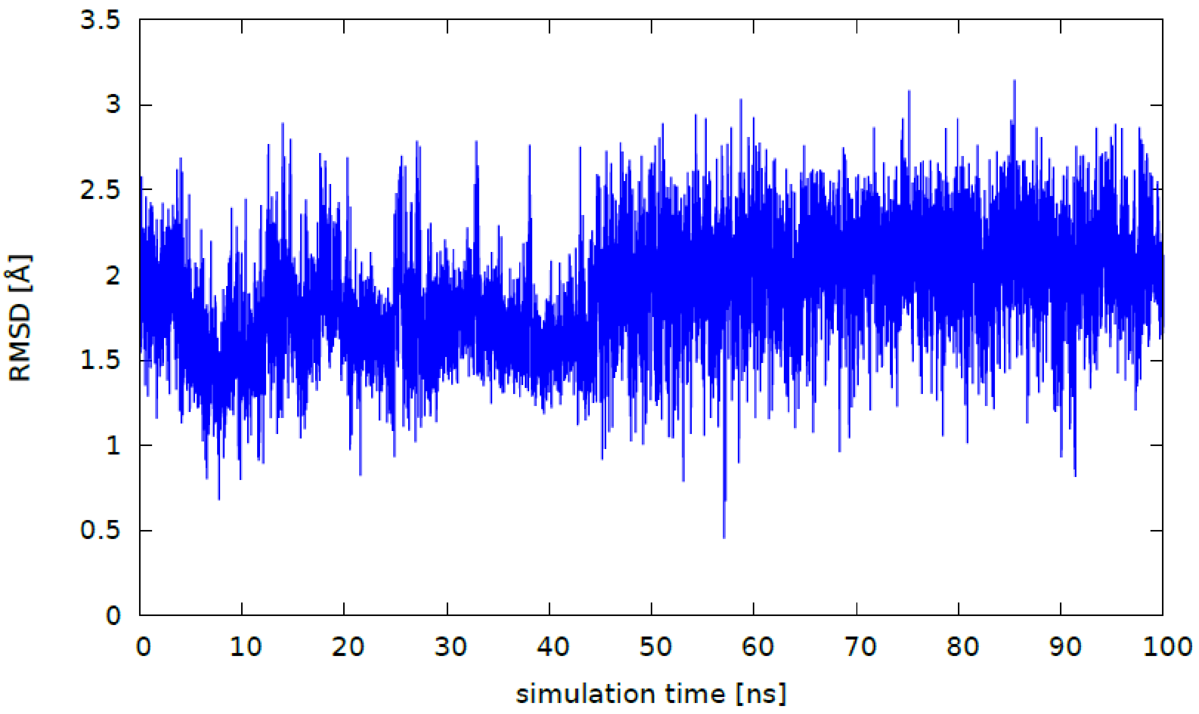

Figure S10. RMSD graph for compound 10.

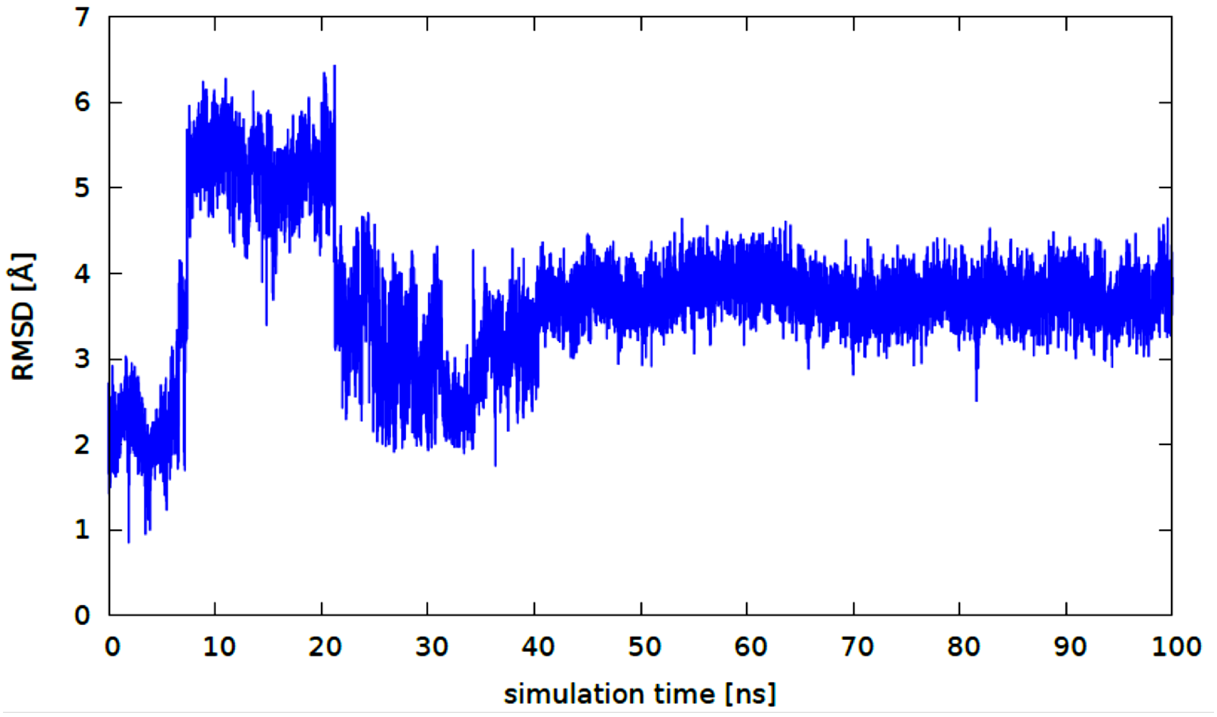

Figure S11. RMSD graph for compound 11.

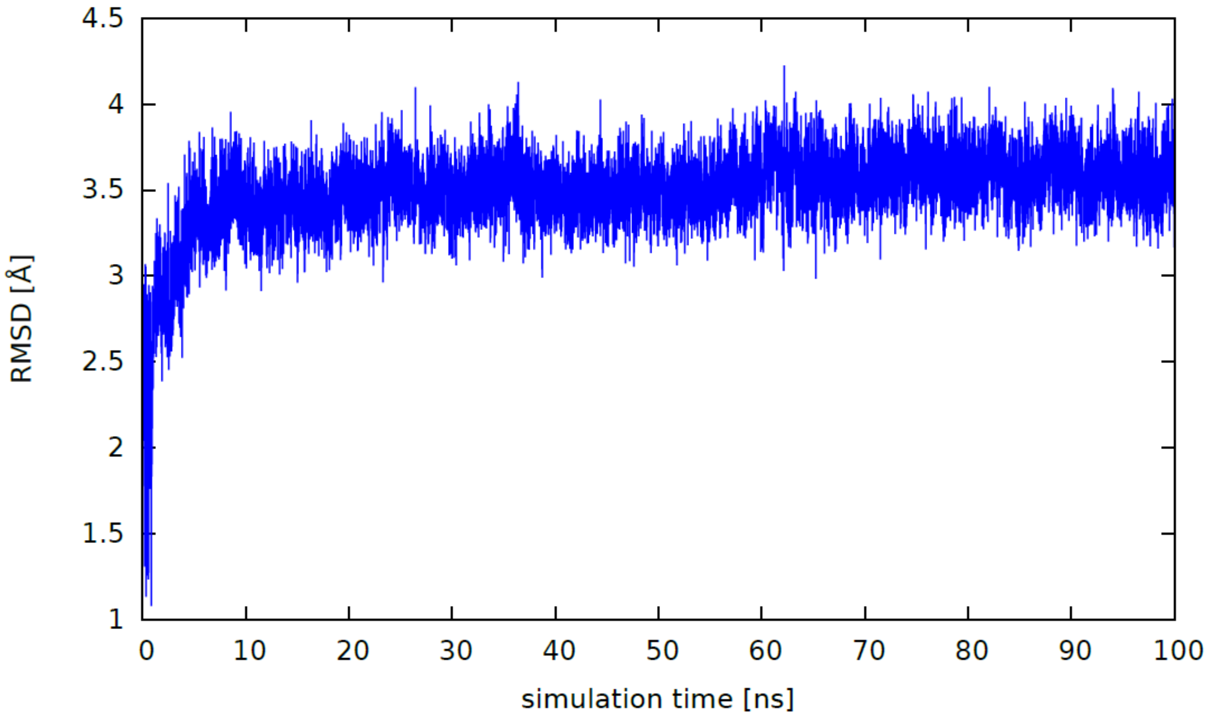

Figure S12. Overview of the interaction pattern obtained with dynophore analysis for compounds 2-5. Calculated percentage of the occurrence of a dynamic pharmacophore element on the basis of all frames broken into element-interacting amino acids pairs.

2

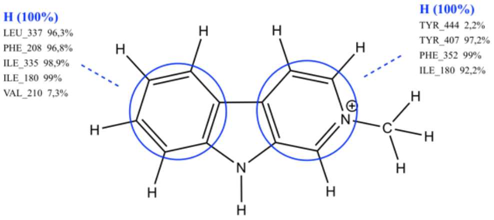

3

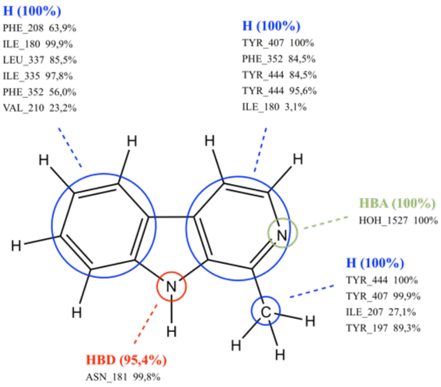

4

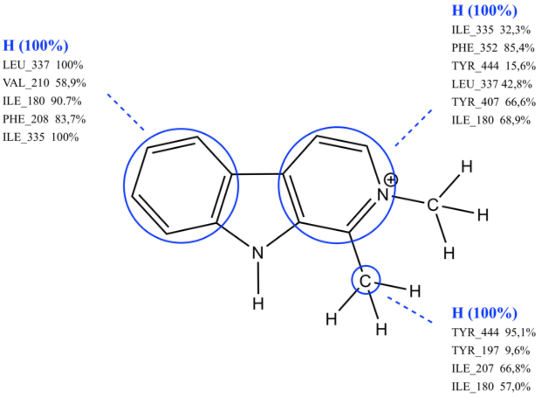

5

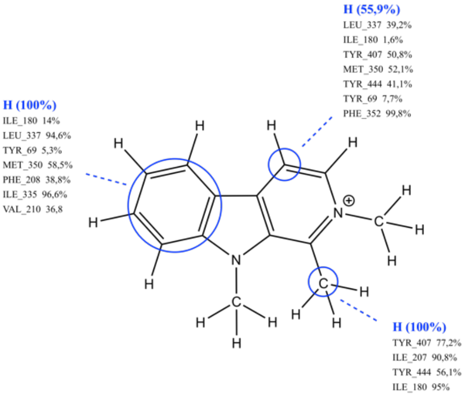

Figure S13. Overview of the interaction pattern obtained with dynophore analysis for compounds 7-9. Calculated percentage of the occurrence of a dynamic pharmacophore element on the basis of all frames broken into element-interacting amino acids pairs.

7

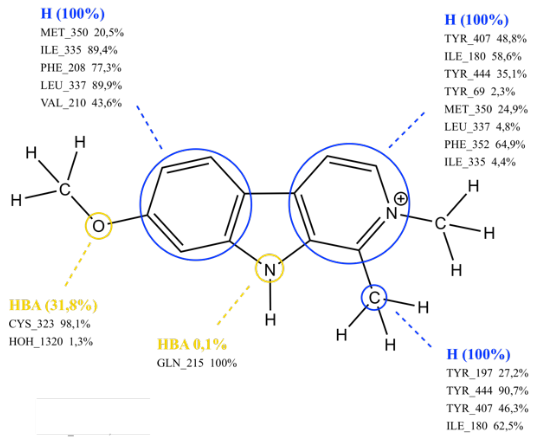

8

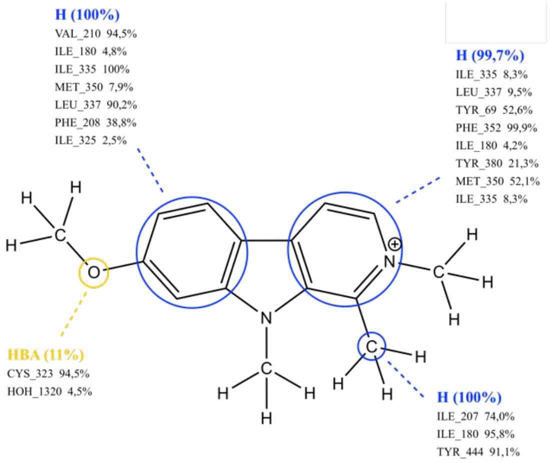

9

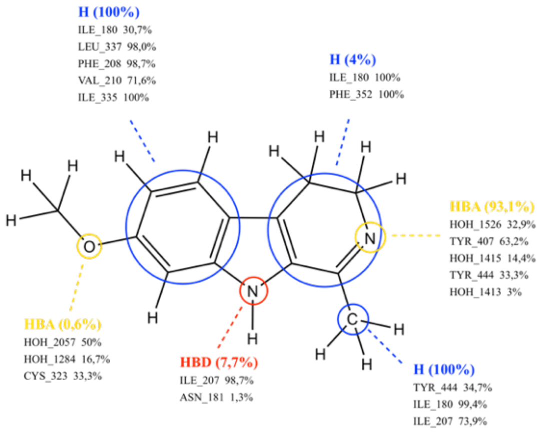

Figure S14. Comparison between X-ray binding pose of harmine (compound 6, in silver) with the determined binding modes using scoring functions ChemScore (in green), GoldScore (in violet) and ChemPLP (in red).

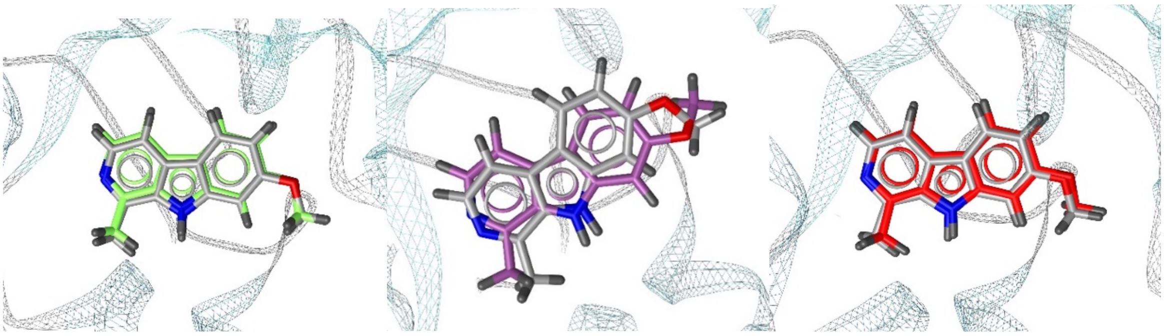

Table S12. The average number of water molecules present in the MAO-A active site (i.e., within 5 Å of the pertinent compound) during the molecular dynamics simulation, with standard deviations also provided.

Compound	Structure	Average num. of water molecules	ST. DEV.
**1**	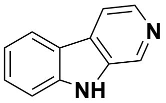	6.1	2.1
**2**	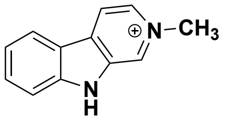	7.4	1.1
**3**	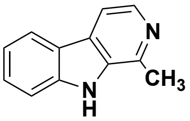	4.3	0.9
**4**	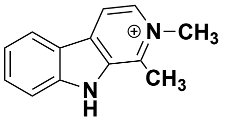	3.4	1.0
**5**	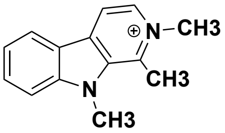	4.9	0.9
**6**	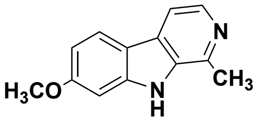	4.9	0.9
**7**	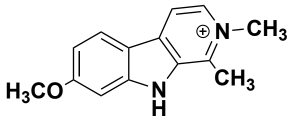	6.2	1.1
**8**	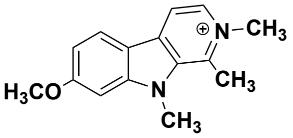	4.3	0.9
**9**	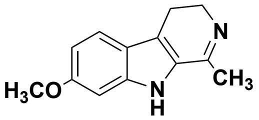	2.5	0.6
**10**	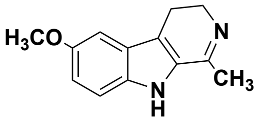	6.1	1.3
**11**	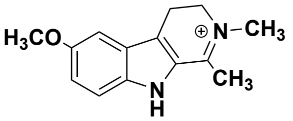	5.2	1.6
